# Impact of supersaturation on growth, critical radius, and size in neomycin nanoparticle crystallization using anti-solvent and CTAB

**DOI:** 10.1039/d5ra08649c

**Published:** 2025-12-03

**Authors:** Sirous Motahari, M. Reza Malayeri, Mehrdad Manteghian

**Affiliations:** a Department of Chemical Engineering, School of Chemical and Petroleum Engineering, Shiraz University Shiraz Iran malayeri@shirazu.ac.ir; b Department of Chemical Engineering, Faculty of Engineering, Tarbiat Modares University Tehran Iran

## Abstract

This study investigates the controlled synthesis of neomycin nanoparticles *via* antisolvent-induced crystallization using a water-acetone system with cetyltrimethylammonium bromide (CTAB) surfactant as a stabilizer. Comprehensive analyses, including SEM, TEM, DLS, EDS, TGA, DSC, XRD, AFM, and FT-IR, confirmed the production of nanoparticles with uniform morphology and a size distribution ranging from 22 to 265 nm. Kinetic studies revealed that higher supersaturation profoundly reduced induction time and led to the formation of nanoparticles with smaller critical sizes. Nucleation mechanism analysis based on classical nucleation theory indicated the dominance of homogeneous primary nucleation under high supersaturation conditions, directly impacting key parameters such as critical nucleus radius, crystal growth rate, and final nanoparticle size. The results also showed noticeable changes of the interfacial energy in the presence of CTAB, resulting in higher colloidal stability. Moreover, CTAB broadened the metastable zone width (MSZW), enabling more precise control over the crystallization process. Additionally, the simultaneous optimization of process parameters, including supersaturation degree, agitation rate, and CTAB concentration, achieved nanoparticles with optimal physicochemical properties. These findings represent a significant step in the development of nanopharmaceutical formulations with controlled release and enhanced therapeutic efficiency.

## Abstract 2

Neomycin nanoparticles with a bimodal size distribution (22 to 265 nm), were synthesized using a CTAB-mediated antisolvent crystallization process by controlling supersaturation and induction time.

## Graphical abstract

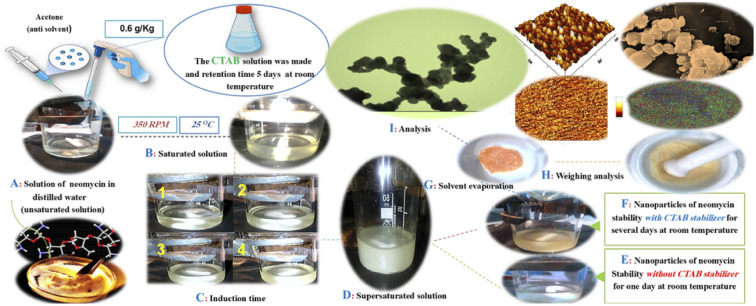

## 1. Introduction

Neomycin is a broad-spectrum aminoglycoside antibiotic widely used to treat skin and intestinal bacterial infections. This is mainly due to its effectiveness against both Gram-positive and Gram-negative bacteria, as it inhibits protein synthesis to exert its antibacterial effect.^1–3^ Its popularity stems from its availability and low cost, though its conventional forms are limited by poor solubility and systemic toxicity, even when used in combination with other agents.^4,5^

To overcome these limitations, nanoparticulate formulations have emerged as a promising strategy for antibiotic delivery, offering enhanced targeting and reduced dosing frequency. Nevertheless, achieving precise control over nanoparticle size and distribution remains a significant challenge, as these parameters critically influence drug release kinetics and cellular uptake.^6^ Nanoparticle production methods are generally categorized into top-down and bottom-up approaches.^7,8^ Among bottom-up nanoparticle production methods, which involve the assembly of nanoparticles from molecules, antisolvent crystallization has gained interest in the pharmaceutical industry.^9–11^ This method is ideal for poorly soluble drugs like neomycin, enabling precise control over particle size and distribution to produce particles with tailored properties. This enhances drug solubility, dissolution rate, and bioavailability.^9–12^ The process is driven by supersaturation, where higher levels promote rapid nucleation (yielding smaller particles) and lower levels lead to slower growth and larger crystals.^13–15^

Optimizing this balance is therefore essential to reduce polydispersity and improve reproducibility. According to classical nucleation theory, the critical radius, the minimum stable size of a nucleus, is inversely related to the degree of supersaturation,^16^ further reinforcing the need to precisely control its impact on the final particle size.

In antisolvent crystallization, highly relevant for a poorly soluble antibiotic like neomycin, a miscible nonsolvent is introduced to decrease solubility, thereby inducing this crucial supersaturation, and triggering nucleation.^17,18^ This involves a two-stage process: an initial phase separation where crystals begin to form, followed by crystal growth.^19,20^ The introduction of the antisolvent drives the system into a metastable, supersaturated state, and the extent of the achieved supersaturation profoundly influences the nucleation rate and induction time, ultimately dictating the final particle size and morphology.^21–24^

Theoretically, increased supersaturation reduces the critical radius, promoting the formation of smaller nanoparticles.^25,26^ However, excessively high supersaturation can induce secondary nucleation or Ostwald ripening,^27^ while the complex structure and high polarity of antibiotics like neomycin may lead to aggregation or amorphous phase formation.^28^ These challenges are exacerbated by the inherent agglomeration tendency of nanoparticles.^29,30^ Consequently, precise control over solvent/antisolvent selection, agitation rate, and supersaturation is crucial for obtaining uniform neomycin nanocrystals,^31^ which is why stabilizers are commonly employed in antisolvent precipitation to prevent aggregation and ensure colloidal stability. Following section discusses pertinent studies on antisolvent crystallization of antibiotic nanoparticles, thereby contextualizing the present work on neomycin.

The recrystallization of caffeine was studied by Torkian *et al.*,^32^ who found that at high supersaturation, the induction time becomes less dependent on supersaturation due to the increased significance of growth kinetics and secondary nucleation. Cheng *et al.*^33^ investigated the effects of polymers like PVP on indomethacin, showing that PVP exhibits a strong inhibitory effect on crystal growth, potentially by delaying drug molecule integration onto the crystal surface.

The synthesis of neomycin nanoparticles *via* induced crystallization using PVP as a stabilizer was investigated by Motahari *et al.*^29^ Using an *in situ* turbidimeter for induction time measurements, they observed a bimodal size distribution from 17 to 235 nm and applied classical nucleation theory to confirm primary nucleation as the dominant process. Kumar *et al.*^34^ have conducted a comprehensive review on antisolvent crystallization, emphasizing its role in enhancing solubility, dissolution rate, and bioavailability of pharmaceuticals, while also highlighting innovative approaches like ultrasound. An average particle size of 320 nm for telmisartan nanoparticles was achieved by Sharma *et al.*^35^ through the optimization of process parameters, including drug concentration, antisolvent volume, injection rate, and temperature.

Yu *et al.*^36^ investigated the impact of stirring speed and feed rate on paracetamol crystal aggregation, noting that higher agitation reduced aggregation of larger particles, while increased feed rate worsened aggregation due to enhanced nucleation. Advanced methods for producing spherical crystals of clopidogrel bisulfate were developed by An *et al.*,^37^ demonstrating that their approach minimized antisolvent volume while enhancing process control. A comprehensive investigation of sodium sulfate decahydrate crystallization by Khaleghi *et al.*^38^ revealed that aluminum nitride particles significantly enhanced nucleation kinetics by reducing induction time and metastable zone width without altering solubility.

**Fig. 1:**
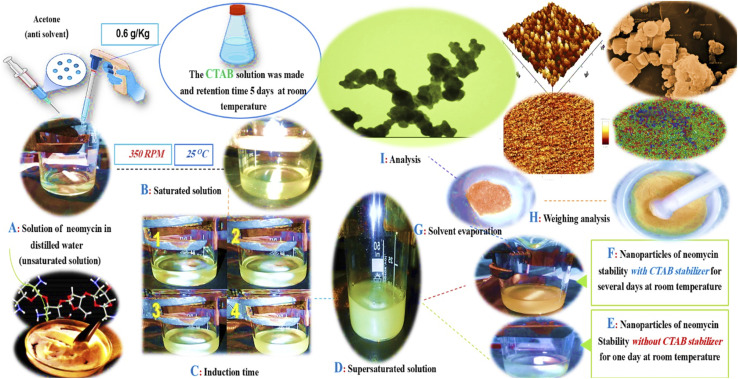
The solution preparation to final results would include several consecutive stages: (A) unsaturated solution, (B) saturation state, (C) induction time measurement, (D) final supersaturation, (E) stability without CTAB, (F) stability with CTAB, (G) solvent evaporation, (H) weighing, and (I) nanoparticle characterization.

Despite significant advances in nanoparticle engineering, the interplay between supersaturation, critical radius, and growth kinetics in neomycin formulations remains underexplored, with existing literature focusing more on solubility and toxicity than fundamental crystallization mechanisms.^39,40^ This study aims to address this gap by examining supersaturation's role in the antisolvent crystallization of neomycin nanoparticles. It is hypothesized that optimizing supersaturation parameters with CTAB as a stabilizer will produce nanoparticles with improved stability and properties. To verify this, the effects of supersaturation on crystallization kinetics and nucleation mechanisms will be investigated, CTAB's stabilization efficacy will be assessed, and the nanoparticles will be characterized using FESEM, TEM, XRD, DLS, FTIR, and thermal analyses. The experimental setup is shown in Fig. 1.

## 2. Materials and experimental procedure

### 2.1. Materials

The neomycin sulfate (99.8%) was purchased from Sigma-Aldrich. CTAB and acetone (CH_3_COCH_3_, 99.9%) were provided from Merck.

### 2.2. Material characterization

The samples were comprehensively characterized using a suite of analytical techniques. Morphology and elemental composition were investigated using ZEISS instruments: field emission scanning electron microscopy (FESEM, Sigma VP model) equipped with energy-dispersive X-ray spectroscopy (EDX-b), and transmission electron microscopy (TEM, EM10C-100KV model). Particle size distribution was analyzed by dynamic light scattering (DLS) using a Malvern Zetasizer. Thermal properties were assessed using Mettler Toledo equipment: thermogravimetric analysis and derivative thermogravimetry (TGA/DTG, TGA2 model) for stability, and differential scanning calorimetry (DSC, DSC-2 model) for thermal transitions. The crystalline structure was determined by X-ray diffraction (XRD, Panalytical X'Pert Pro model), and functional groups were identified by Fourier-transform infrared spectroscopy (FT-IR, PerkinElmer Spectrum Two model). Surface topography was evaluated by atomic force microscopy (AFM, Bruker JPK Nanowizard-2 model). And also Mapping and EDX-a was done by Vega 3 model aof TESCAN Company.

### 2.3. Preparation of CTAB solution

To prepare the CTAB solution, 0.365 g of CTAB powder (molecular weight = 364.45 g mol^−1^) was dissolved in 1.0 liter of double-distilled water and stirred thoroughly to achieve an aqueous solution with a concentration of 1.00151 mM. The addition of CTAB is hypothesized to reduce the surface tension of the formed nanoparticles, a phenomenon attributed to its significant surfactant properties.^39,40^ This solution was stored at room temperature for five days and stirred twice daily (morning and evening) to ensure homogeneity and stability. The final CTAB solution was subsequently utilized as a stabilizing agent in the synthesis of neomycin nanoparticles.

### 2.4. Synthesis of neomycin nanoparticles

**Fig. 2:**
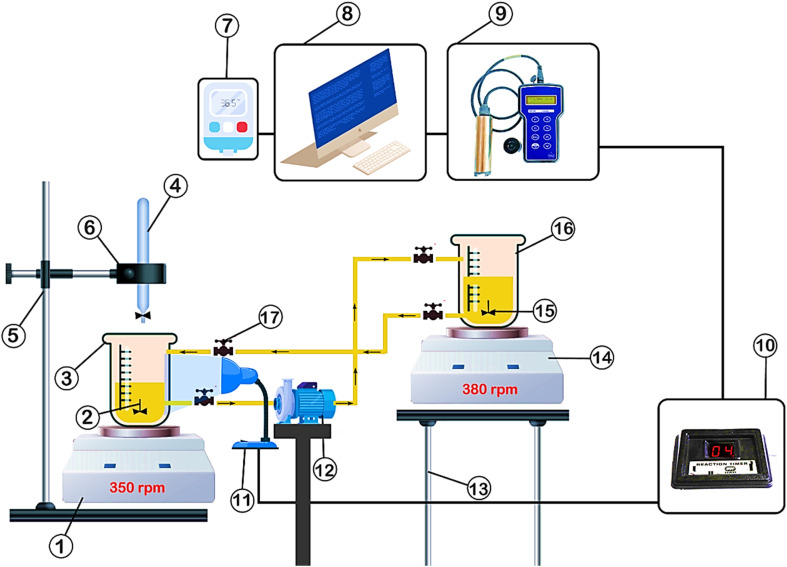
Schematic diagram of the laboratory setup: (1) first stirrer, (2) mixer or (magnetic stirrer), (3) first crystallizer, (4) burt, (5) base, (6) laboratory clamp, (7) thermometer, (8) Pc Lab, (9) *In situ* turbidity meter, (10) reaction timer and (11) LED, (12) pump, (13) support, (14) second stirrer, (15) mixer or (magnetic stirrer), (16) second crystallizer and (17) valve.

In this section, the method for the synthesis of neomycin nanoparticles is explained in detail. First, an exact amount of neomycin powder (purity ≥ 95%) was weighed using a digital analytical balance (accuracy: ±0.1 g) based on the desired equilibrium concentration. The weighed neomycin was then added to 10 g of double-distilled water (25 °C). To ensure complete dissolution, the mixture was homogenized using a magnetic stirrer at controlled speeds of 300, 350, 400, and 450 rpm for 20 minutes. This range of stirring speeds was selected to investigate the effect of shear force on solution homogeneity. After confirming the homogeneity of the neomycin solution, a predetermined amount of CTAB surfactant with a concentration established from prior studies was added to the beaker. To achieve optimal mixing, the stirring system was activated at a constant speed between 300 and 450 rpm, and the mixing process continued until equilibrium was reached. The solution temperature was continuously monitored using a calibrated digital thermometer (±0.1 °C). A turbidity sensor was installed 2 cm from the crystallizer wall to measure changes in turbidity. Pure acetone (HPLC grade) was added dropwise (0.02 mL per drop) at 5-minute intervals. The first signs of physical change (*e.g.*, localized cloudiness) were recorded as the initial saturation point. Acetone addition continued until stable turbidity (supersaturation) was achieved under isothermal conditions (25 °C). The induction time (the interval between initial saturation and supersaturation), as clearly depicted in Fig. 1, Section C (the induction time segment of steps 1 to 4), was measure using a synchronized turbidimeter and reaction timer data. To ensure reproducibility, the entire process was repeated in four independent trials under controlled conditions (constant temperature, humidity, and lighting). The schematic diagram of the used laboratory setup is shown in Fig. 2.

It should be noted that, to mitigate the totoxicity of CTAB, the surfactant was extensively removed post-synthesis. The crude nanoparticle suspension was subjected to centrifugation (12 000 rpm, 30 min, 4 °C) to eliminate free CTAB and acetone. The precipitate was then rigorously washed *via* five cycles of redispersion and centrifugation in a cold ethanol–water solution (70 : 30 v/v, 4 °C) to disrupt CTAB's hydrophobic interactions, followed by a final wash with cold deionized water. Efficacy of CTAB removal was quantitatively confirmed by EDS analysis, which showed a 96% reduction in bromine content (a signature element of CTAB) from 48.5% (Fig. 6a, before washing) to 2% ((Fig. 6b, after washing) after the washing process.

### 2.5. Effect of dynamic shock velocity

In this study, the rotational speed of the crystallizer was adjusted to different values (300, 350, 400, and 450 rpm), which differed from our previous work that employed a different stabilizer.^29^ To enhance the mixing between the solvent (water), solute (neomycin), and antisolvent (acetone), a novel approach, aimed at process intensification, was implemented by progressively increasing the agitation rate at the moment of acetone droplet addition. In this method, the agitator speed was linearly increased over 30 seconds after the addition of acetone droplets (antisolvent) under near-saturation conditions, from 300 to 330, 350 to 380, 400 to 430, and 450 to 480 rpm. This approach facilitates two key mechanisms: first, the increased speed enhances shear force, preventing the formation of localized high-concentration regions (hotspots) around the acetone (antisolvent) droplets. Second, the higher agitation speed improves mass transfer, promoting a more uniform distribution of the antisolvent in the aqueous phase and enabling more efficient penetration of acetone into the solvent–solute matrix. Consequently, this sudden increase in agitator speed would greatly influence the process kinetics and is defined as the dynamic shock velocity. This phenomenon accelerates nucleation by inducing turbulence at the water (solvent)/acetone (antisolvent) interface. The dynamic shock velocity can enhance mixing and supersaturation stability by reducing induction time. However, in some cases, it may delay crystal nucleation by prolonging induction time and reducing localized heterogeneities.^41–44^

### 2.6. Measurement of induction time

Induction time represents a key parameter in crystallization processes, being predominantly influenced by three factors of supersaturation, temperature, and agitation rate.^29,32^ This parameter characterizes the time interval between the onset of initial physical changes (saturated state) and the complete formation of a turbid phase (supersaturated state) within the system. Investigations indicate that both the optimal agitation speed and the application of dynamic shock have a significant influence on the system's supersaturation level.^29,33,35,36^ A sudden increase in agitation rate initially induces localized supersaturation reduction through turbulence generation at the interface between unstable and metastable zones. The analysis reveals a three-stage induction mechanism influenced by supersaturation and CTAB, as stabilizer. CTAB modulates the process by reducing surface energy and stabilizing subcritical clusters, which shortens the nucleation time and broadened the metastable zone. Agitation dynamics initially cause a temporary decrease in localized supersaturation (dynamic shock), followed by a stabilization phase that enables controlled crystal growth.

Methodologically, induction time was measured using both visual observation and *in situ* turbidimetry. The results confirm that turbidimetry provides more accurate and reproducible data due to its ability to detect early nucleation events before they become visually apparent, minimizing human error.^29,45^ This establishes in-line turbidimetry as a superior technique for precise crystallization kinetics studies.^46^ The results related to induction time measurement using two methods of visual and *in situ* turbidimeter are presented in Table 1.

**Table 1:** Comparison between the induction time measurement using two methods of visual and *in situ* turbidimeter

Supersaturation	Number of repeats	Induction time (s) (visual method)	Standard deviation	Induction time (s) (turbidimeter method)	Standard deviation
1.18	8	129	10	112	10
1.2	8	117	9	103	9
1.21	8	123	11	101	10
1.25	8	121	10	99	9
1.29	8	115	9	96	8
1.33	8	109	8	94	8
1.34	8	114	9	91	8
1.351	8	101	8	90	7
1.379	8	110	9	89	7
1.408	8	103	10	86	8
1.43	8	102	9	84	7
1.45	8	109	8	83	6
1.47	8	97	8	81	7
1.48	8	93	9	79	7
1.66	8	100	10	77	7
1.663	8	95	8	76	6
1.664	8	99	8	74	6
1.67	8	85	8	73	5
1.785	8	88	7	70	6
1.9	8	79	7	64	5

## 3. Results and discussion

### 3.1. Characterization of neomycin nanoparticles

**Fig. 3:**
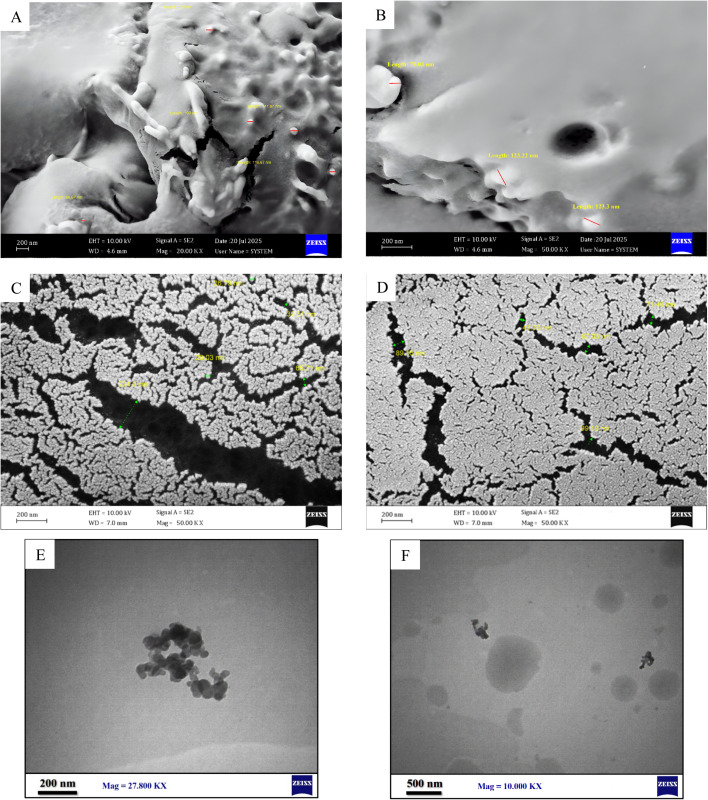
(A) FSEM (20.00 KX), (B–D) FSEM (50.00 KX), (E) TEM (27.800 KX) (F) TEM (10.00 KX) images of nanoparticles without CTAB as stabilizer.

Early attempts to synthesize neomycin nanoparticles without the use of the stabilizer CTAB resulted in the formation of nanoparticles with improper size and morphology, as well as agglomeration of the nanoparticles, as clearly demonstrated by FSEM/TEM analyses in Fig. 3.^29,47^ This colloidal instability can be rationalized through DLVO theory, where the absence of ionic stabilizers leads to the predominance of van der Waals forces over electrostatic repulsion - a phenomenon particularly pronounced in high surface-to-volume ratio nanoparticles.^48^ To achieve nanoparticles with controlled morphology and improved stability, the cationic surfactant CTAB was employed, which operates through three primary stabilization mechanisms: (1) electrostatic stabilization *via* adsorption of quaternary ammonium groups that enhance surface zeta potential, (2) steric stabilization through hydrocarbon chains forming a protective barrier, and (3) surface interactions where neomycin's hydroxyl groups establish hydrogen bonding with CTAB's polar groups. Characterization results demonstrated that CTAB stabilized nanoparticles exhibited superior size distribution and morphological characteristics. These findings are consistent with previously reported principles of antibiotic nanoparticle stabilization and confirm CTAB's efficacy in preventing particle aggregation while maintaining crystalline structure integrity.^49^

**Fig. 4:**
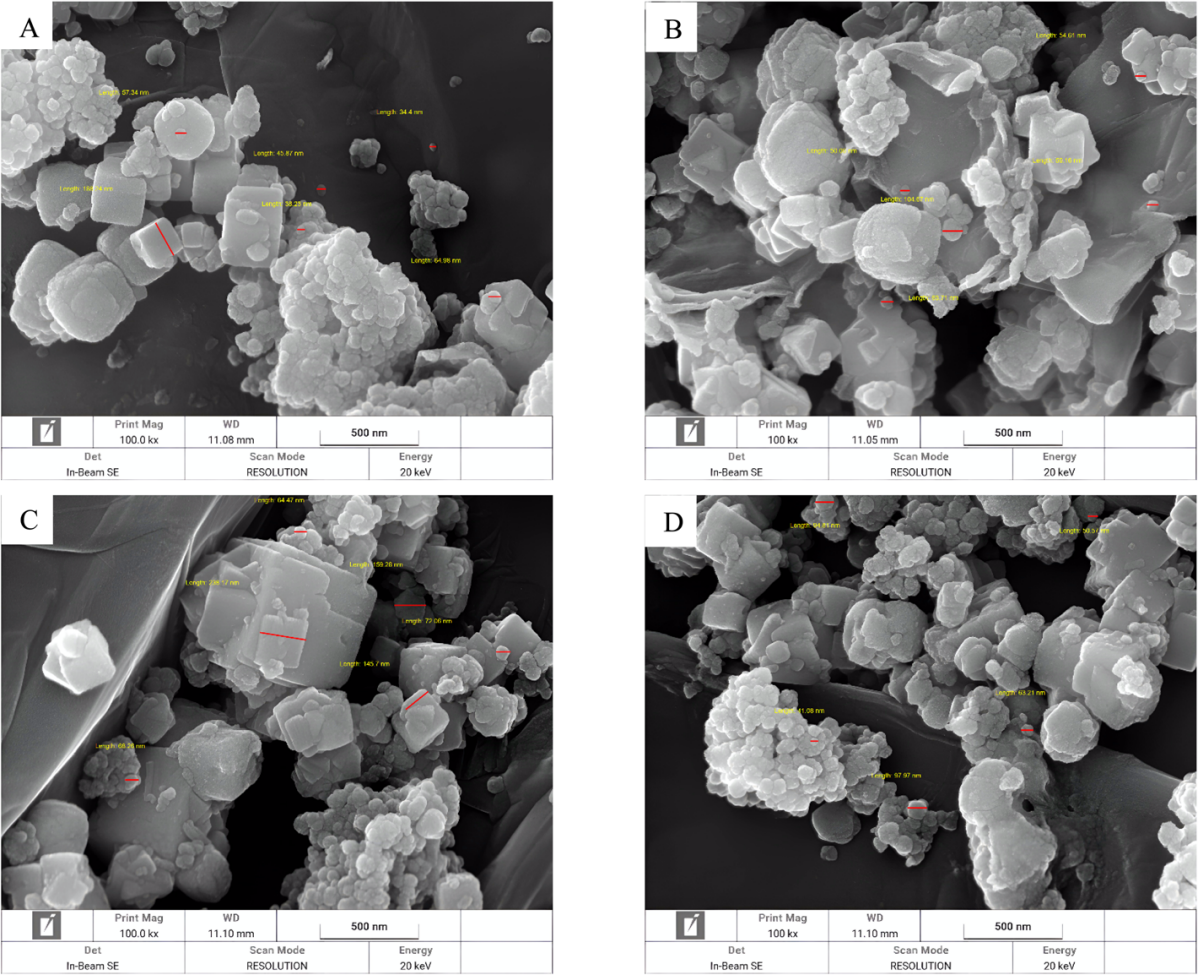
FSEM images of synthesized neomycin nanoparticles (A–D) 100.00KX.

**Fig. 5:**
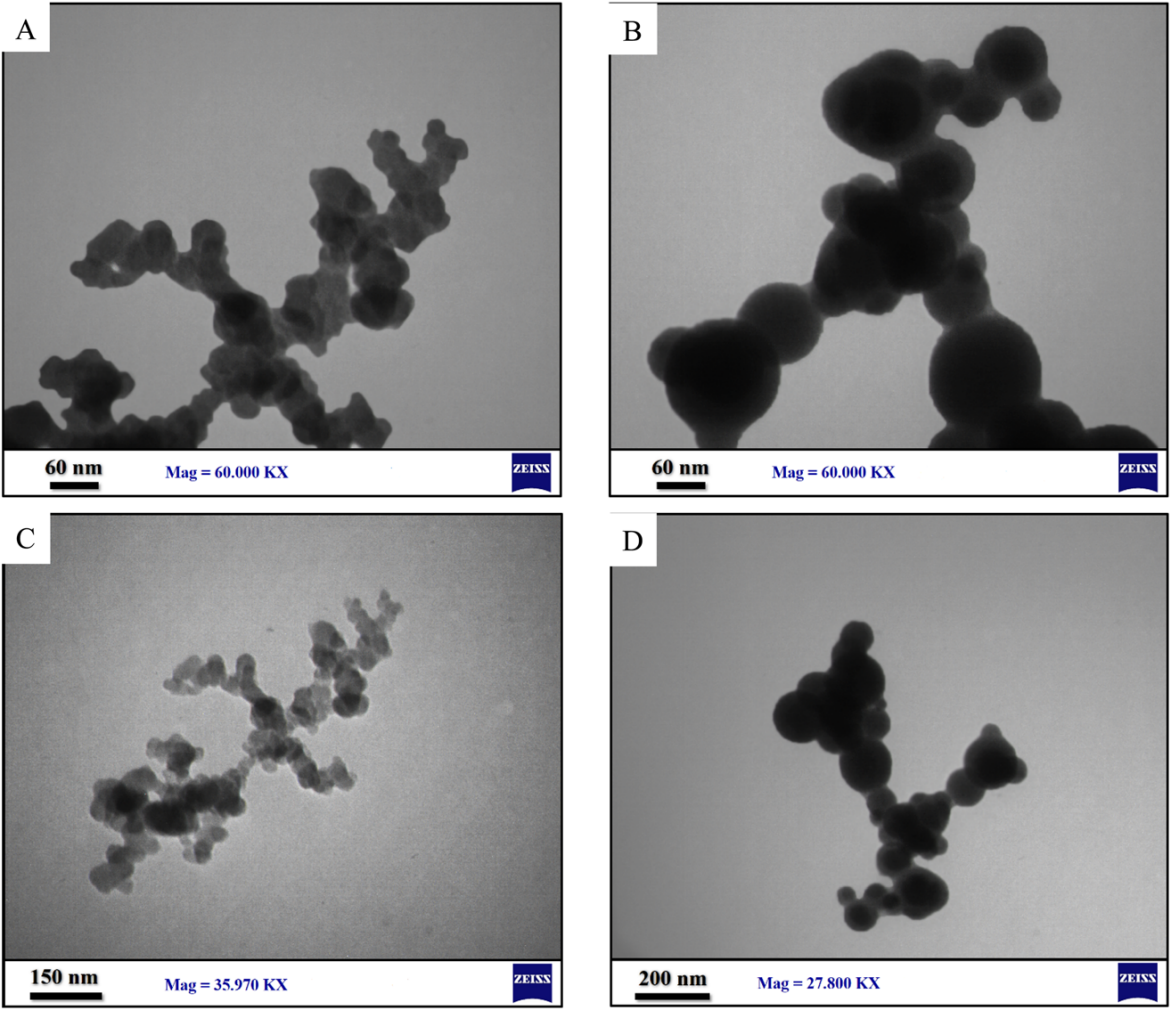
TEM imaging of synthesized neomycin nanoparticles (A and B) 60.00 KX, (C) 35.790 KX and (D) 27.800 KX.

**Fig. 6:**
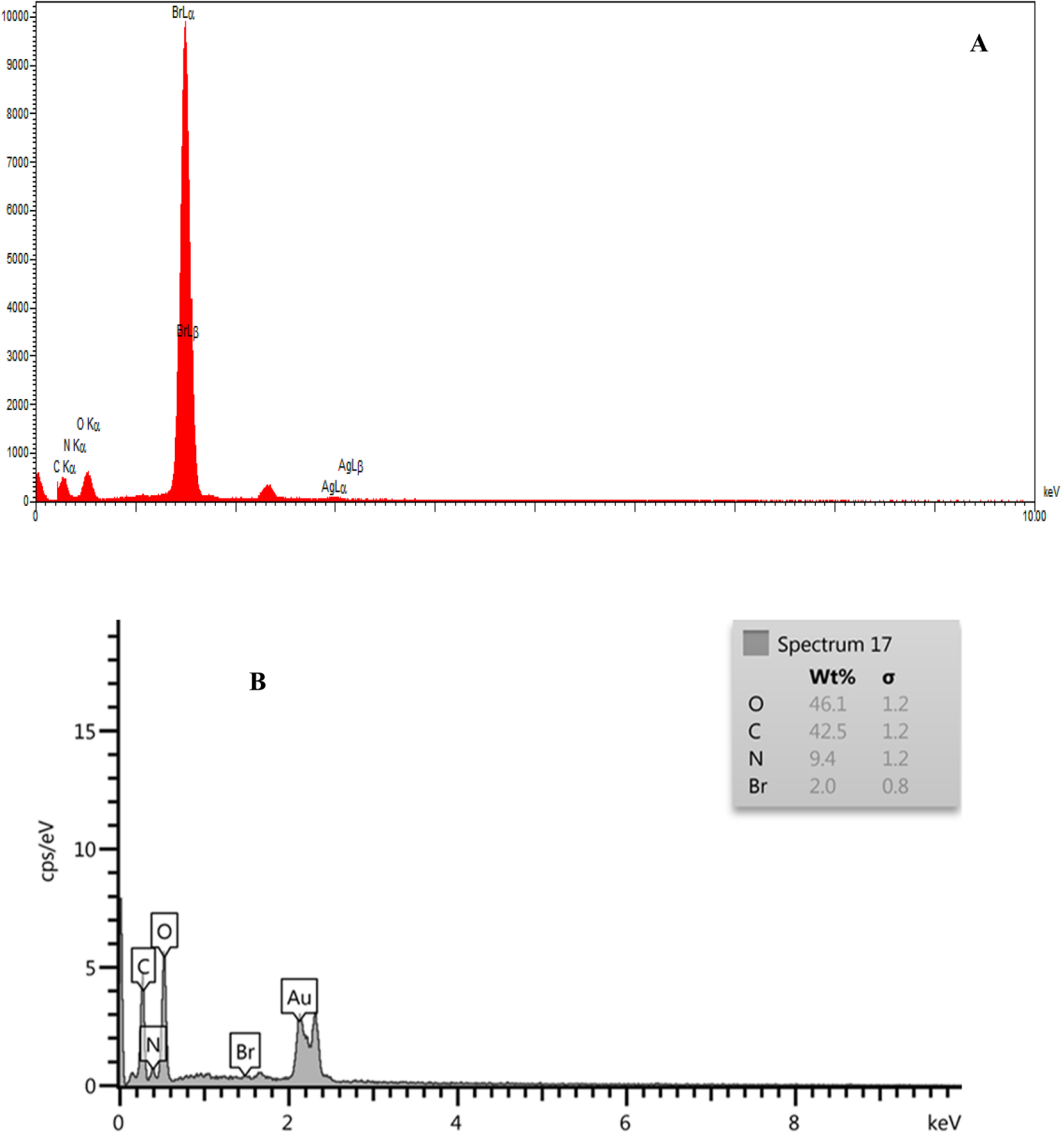
EDX spectrum of neomycin nanoparticles: (A) before CTAB removal, (B) after CTAB removal.

**Fig. 7:**
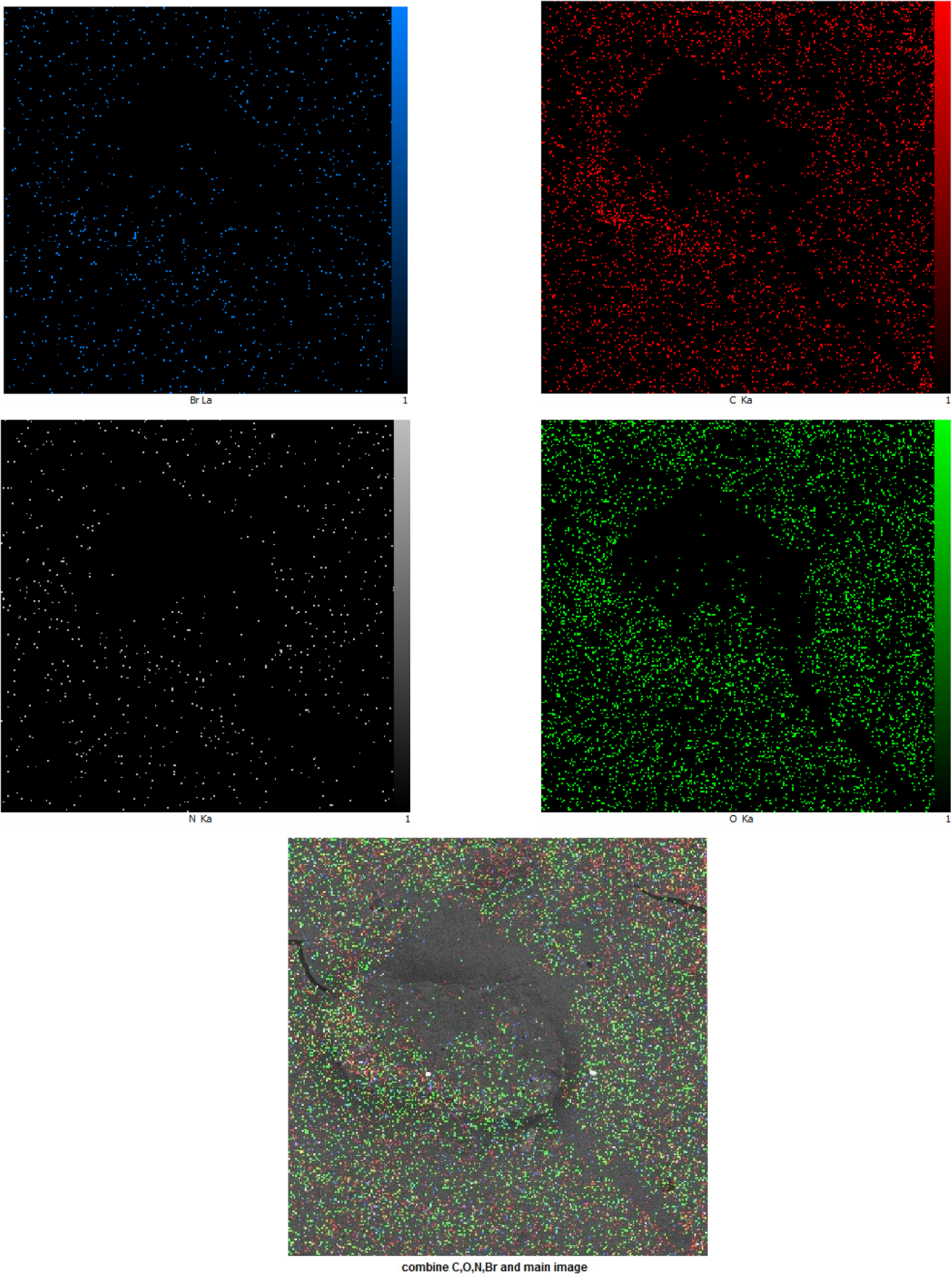
Elemental mapping of synthesized neomycin.

The synthesized CTAB stabilized neomycin nanoparticles were comprehensively characterized using, FSEM, TEM, MAP, FTIR, XRD, AFM, and EDX analyses were conducted analytical techniques. FSEM and TEM images of synthesized neomycin nanoparticles have been presented in Fig. 4 and 5, respectively. FSEM and TEM imaging revealed well-defined morphology and uniform dispersion of the nanoparticles. The EDX spectroscopy and elemental mapping of synthesized nanoparticles were presented in Fig. 6 and 7, respectively. EDX spectroscopy and elemental mapping confirmed the presence and homogeneous distribution of constituent elements (C, O, N, and Br from CTAB), while gold signals were attributed to the sample coating process.

**Fig. 8:**
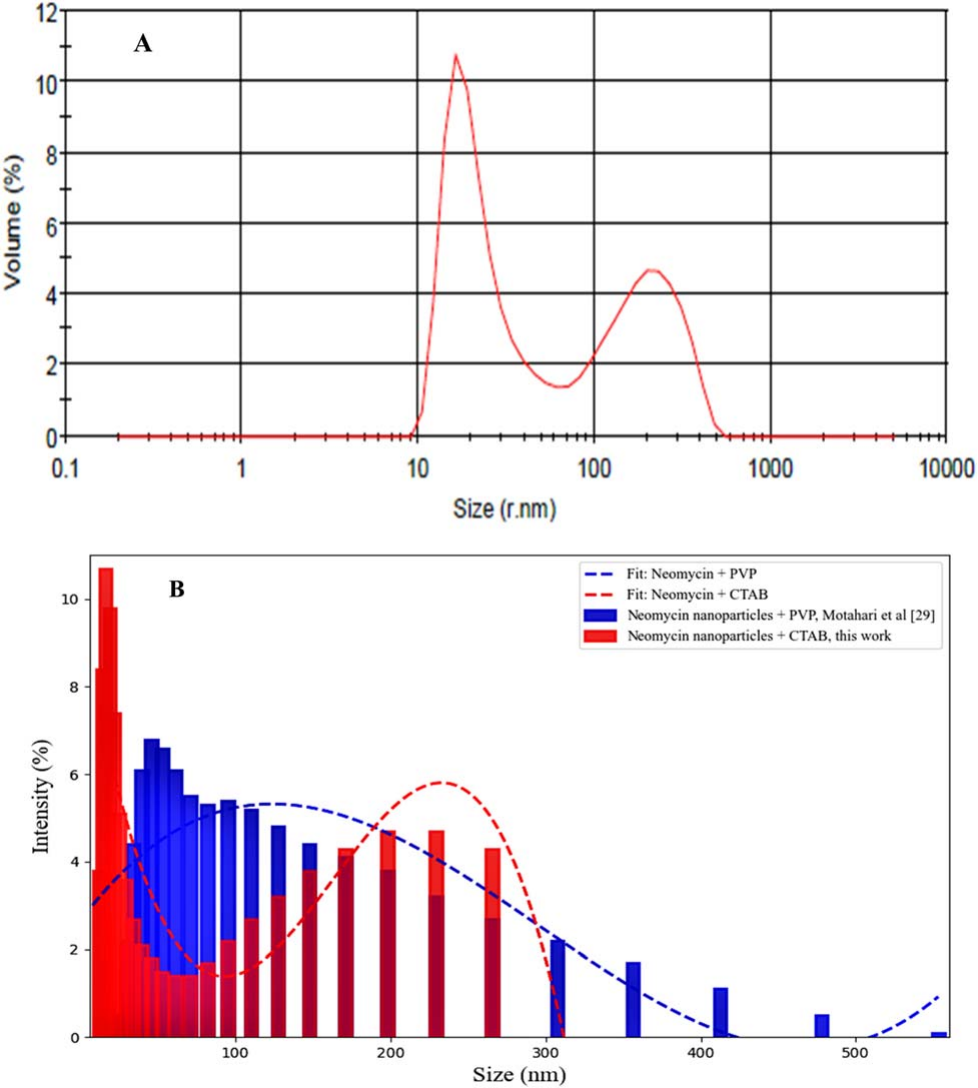
DLS diagram (A) and size distribution (B) for synthesized neomycin nanoparticles.

DLS was employed to determine the nanoparticle size distribution (Fig. 8a). According to the results of DLS analysis along with FESEM and TEM imaging, neomycin nanoparticles with a polydispersity index (PDI) of 0.469 exhibited a bimodal or polydisperse distribution. This characteristic was clearly evident in both the peaks of the DLS profile and the supersaturation values observed in experimental tests.^50^ The observed size distribution in this study can be attributed to the addition of antisolvent (acetone), supersaturation conditions, and the dynamic nature of crystal growth (Fig. 8b). These results indicate that acetone, acting as an antisolvent, affects system saturation and leads to the formation of two distinct crystal size distributions.^51,52^ A comparative study on the stabilizing effects of PVP^29^ and CTAB demonstrated that CTAB-stabilized nanoparticles, similar to those synthesized with PVP,^29^ also exhibit a bimodal distribution. However, the stabilization mechanisms in these two systems are fundamentally different. PVP provides steric stabilization by forming a polymeric layer on the nanoparticle surface, thereby preventing aggregation.^53^ In contrast, CTAB plays a vital role in forming smaller and more stable nuclei through electrostatic repulsion, ensuring colloidal stability *via* its positive surface charge.^54^ Nevertheless, heterogeneity in surface charge distribution may contribute to the formation of larger particles.^55,56^

The mean particle size of PVP-stabilized neomycin nanoparticles was 107.5 nm with a size distribution ranging from 17 to 235 nm,^29^ whereas the CTAB-stabilized nanoparticles in this study showed an average size of 124.7 nm with a distribution range of 22 to 265 nm. These particle size ranges were confirmed by DLS, FESEM, and TEM analyses, verifying the successful formation of neomycin nanoparticles.

**Fig. 9:**
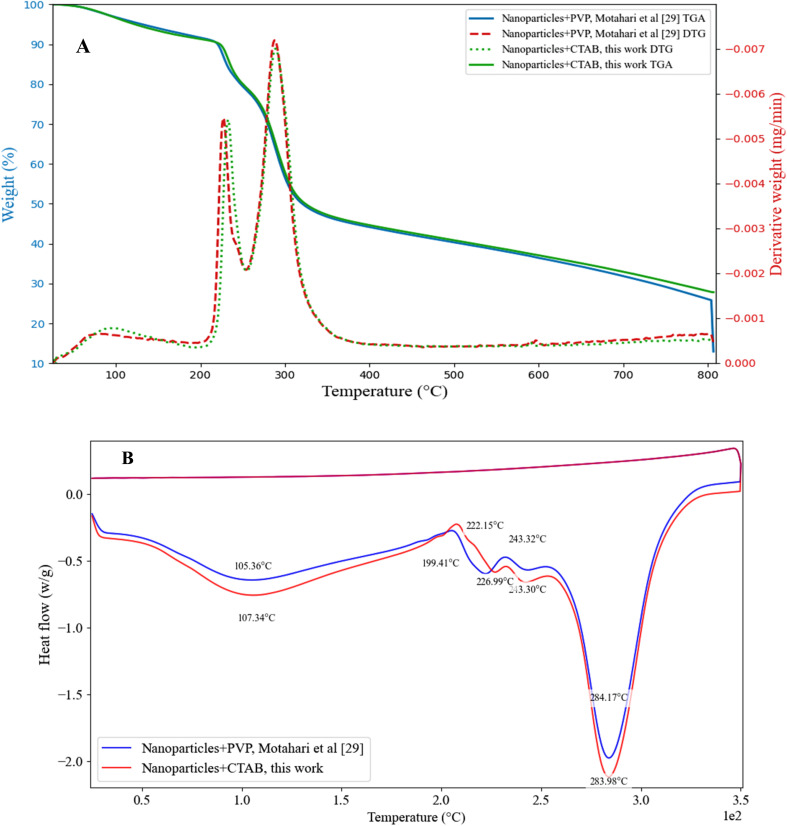
TGA (A), DSC (B) spectra for synthesized neomycin nanoparticles in the presence of CTAB and comparison with existing results.^29^

To evaluate the thermal characteristics of the nanoparticles, TGA and DSC tests were conducted, and the corresponding curves are displayed in Fig. 9a and b. Thermal analysis results demonstrate that the choice of stabilizer significantly influences the thermal behavior of neomycin nanoparticles. Comparative evaluation of two stabilized systems PVP^29^ and CTAB reveals notable differences in their thermal profiles. In the PVP containing system, initial weight loss occurred at 190 °C (8.03%) and 227 °C (11.07%), corresponding to moisture elimination. The CTAB stabilized system showed this weight loss at higher temperatures (200 °C, 8.61% and 230 °C, 10.8%), indicating delayed moisture removal and surface-adsorbed water release. Structural degradation profiles differed markedly: PVP stabilized nanoparticles exhibited decomposition peaks at 250 °C, 288 °C, and 381 °C, while CTAB-stabilized counterparts showed shifted peaks to 250 °C, 290 °C, and 390 °C. This 2–9 °C upward shift in decomposition temperatures clearly demonstrates CTAB's superior thermal stabilization effect. DSC data corroborate these findings, with the melting endotherm appearing at 116.37 °C for CTAB stabilized nanoparticles *versus* 105 °C for PVP-stabilized ones - an 11.37 °C enhancement. These behavioral differences stem from distinct stabilization mechanisms: PVP primarily operates through hydrogen bonding and polymeric coating, leading to relatively abrupt degradation at 288 °C. In contrast, CTAB forms more robust micellar structures through stronger electrostatic interactions, resulting in a more gradual decomposition profile. From an applications perspective, CTAB's higher thermal stability (up to 390 °C) makes it preferable for high-temperature processes, while PVP remains advantageous for pharmaceutical formulations due to its better biocompatibility and lower cost.^57,58^ These findings provide critical insights for rational stabilizer selection in nanoparticle design, particularly for achieving programmed thermal stability in advanced nanomaterial systems. The demonstrated 5–11 °C thermal stability enhancement through CTAB stabilization offers new possibilities for developing thermally robust nanocarriers without compromising structural integrity.

**Fig. 10:**
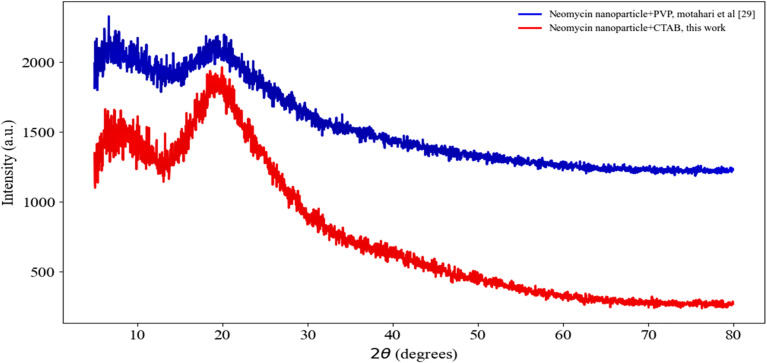
XRD spectra for the synthesized neomycin nanoparticles in the presence of CTAB and comparison with existing results.^29^

The XRD pattern of neomycin nanoparticles in the presence of PVP and CTAB stabilizers reveals significant structural and crystallographic modifications (see Fig. 10). In the presence of PVP, distinct diffraction peaks are observed at 2*θ* = 6.67°, 7.87°, 11.09°, 19.5°, and 20.30°,^45^ while in the presence of CTAB in this study, peaks appear at 2*θ* = 7.509°, 9.351°, 11.357°, 16.817°, 17.883°, 19.937°, and 21.211°. These results indicate the formation of a new crystalline phase in the presence of each stabilizer, which is consistent with previously reported data.^59^ The difference in peak positions between the two systems demonstrates the influence of PVP and CTAB on crystal lattice parameters. Due to its polymeric structure and non-covalent interactions, PVP can alter the inter-crystalline spacing.^60^ On the other hand, CTAB, as a cationic surfactant, may modify the crystal growth pattern through self-assembly mechanisms *via* surface adsorption on nanoparticles.^61^ The presence of these stabilizers can affect the nucleation rate and crystal growth kinetics, where PVP typically inhibits directional growth, leading to the formation of particles with different sizes and morphologies compared to CTAB, as evidenced by TEM and FSEM images. The difference in XRD peak intensities between the two systems indicates changes in crystallinity.^62^ The reduced peak intensity in the presence of CTAB may be due to strong interactions between CTAB and the nanoparticle surfaces. Overall, based on the results of Motahari *et al.* using PVP stabilizer^29^ and the results obtained with CTAB stabilizer in this study, these stabilizers not only influence nanoparticle stability but also modify crystal lattice parameters and crystallization kinetics, leading to the formation of distinct crystalline phases. This is crucial for optimizing the synthesis of nanoparticles with controlled properties.

**Fig. 11:**
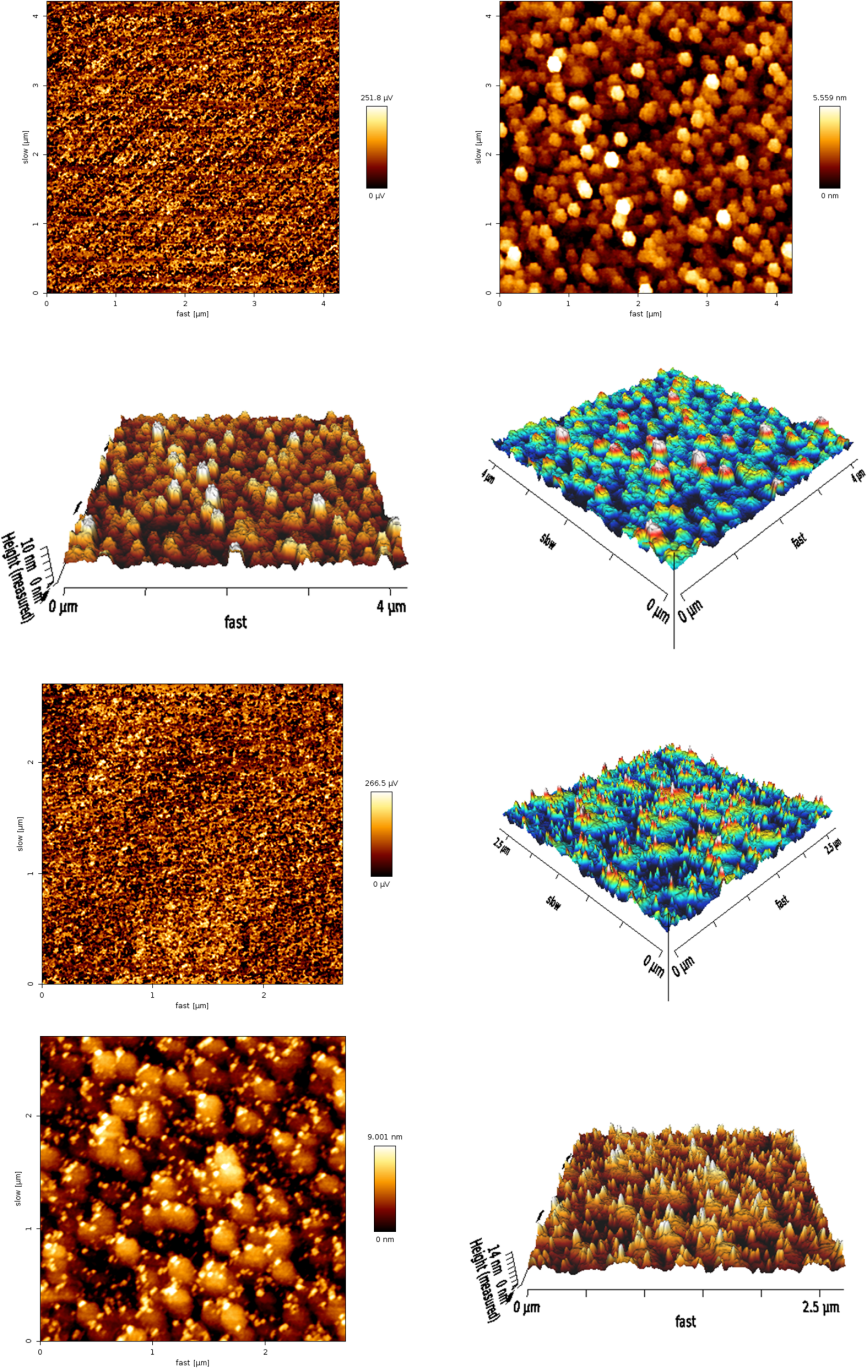
AFM images of the neomycin nanoparticles.

To investigate the surface characteristics of neomycin nanoparticles synthesized in the presence of CTAB stabilizer, three-dimensional atomic force microscopy (3D-AFM) was performed, and the results are presented in Fig. 11a–d. This advanced imaging technique is a powerful tool for detailed examination of crystalline nanostructures, enabling quantitative and qualitative analysis of surface parameters with nanoscale precision.^63^ Three-dimensional AFM analysis allows simultaneous investigation of topographic features in three spatial dimensions (*x*, *y*, and *z*), providing unprecedented insights into crystal growth kinetics and nanoparticle formation mechanisms.^63,64^ The high-resolution images obtained provide essential information about key parameters, including particle size distribution, surface morphology, nanoscale roughness, and the density and distribution of crystalline defects. The results of this study demonstrate that CTAB-stabilized neomycin nanoparticles exhibit suitable crystalline quality, characterized by uniform surface roughness distribution, well-ordered nanostructures, and significantly reduced crystalline defects. These findings are consistent with previous reports on pharmaceutical nanoparticles and studies conducted by Motahari *et al.*^29^ and other reputable research.^65^ Notably, AFM's unique capability to monitor crystal growth dynamics in real time enables a deeper understanding of the influence of surface stabilizers such as CTAB on nucleation and nanoparticle growth mechanisms.^63^ This feature makes AFM an indispensable tool for studying nanocrystal formation. The findings of this research not only confirm the positive effect of CTAB on improving the quality of neomycin nanoparticles, as supported by TEM, FESEM, and XRD results, but also highlight its role in enhancing crystallinity and surface properties for optimized pharmaceutical applications.

**Fig. 12:**
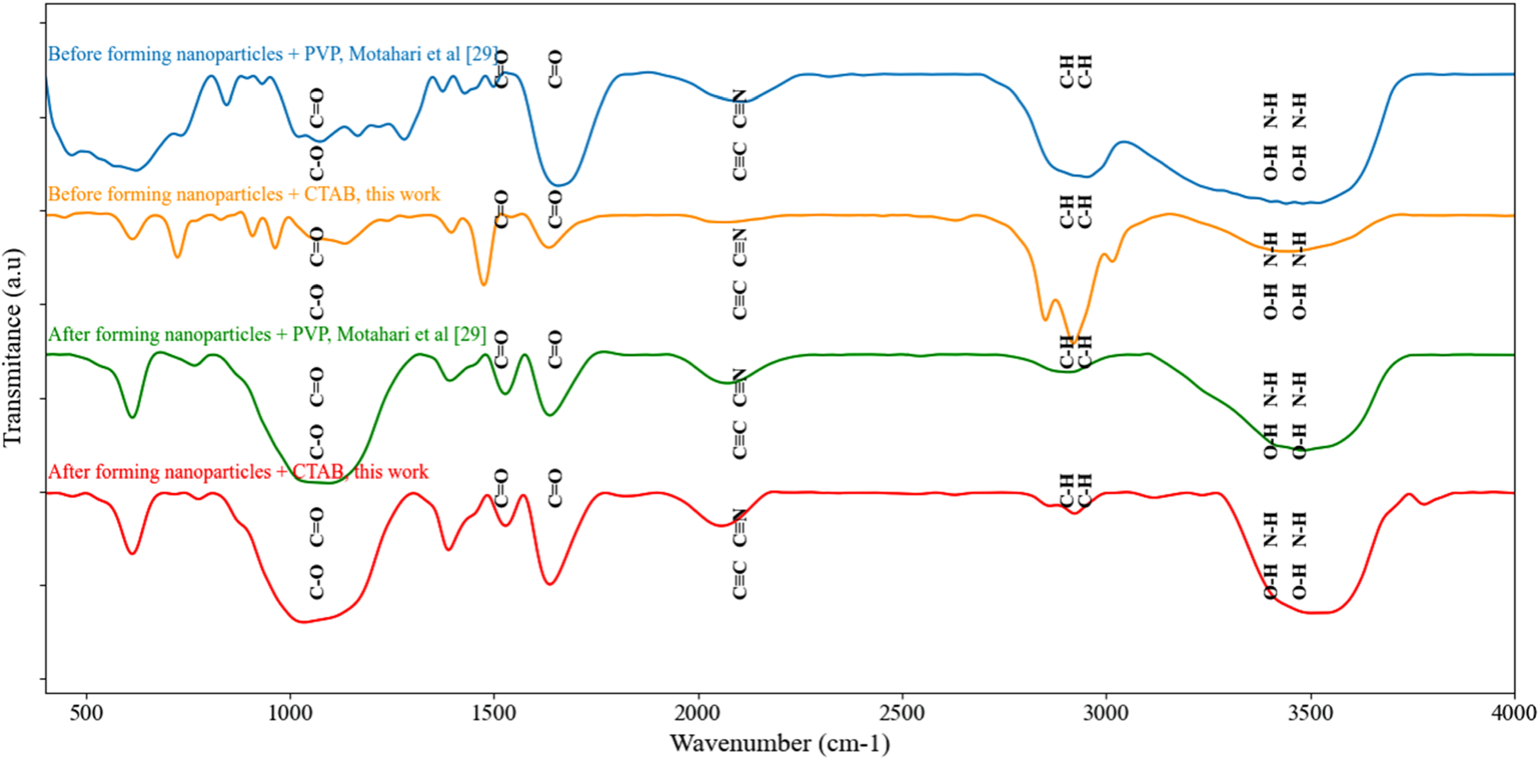
FT-IR spectroscopy before and after neomycin nanoparticles formation in the presence of CTAB and comparison with existing results.^29^

FT-IR spectroscopy was employed as a key analytical tool to investigate structural changes during the formation process of neomycin nanoparticles using two different stabilizer systems (PVP and CTAB) and the results have been presented in Fig. 12.

The spectral data collected during pre and post nanoparticle formation stages provide valuable information about molecular interactions and crystallization mechanisms. In the PVP-stabilized system,^29^ the spectrum of pure neomycin exhibited distinct peaks at 3441 cm^−1^ (O–H/N–H), 2952.86 cm^−1^ (C–H), 1657.30 cm^−1^ (C=O), 1428.13 cm^−1^ (C=C), and 1279.94 cm^−1^ (C–N). Following nanoparticle formation, these peaks shifted to 3482.83, 2908.62, 1637.09, 1391.35, and 1095.2 cm^−1^ respectively, indicating the formation of hydrogen bonds between neomycin's functional groups and PVP's carbonyl units. In contrast, the CTAB-stabilized system exhibited different behavior. The initial spectrum of neomycin with CTAB showed peaks at 3435.90 cm^−1^ (O–H/N–H), 2919.85 cm^−1^ (C–H), 1634.30 cm^−1^ (C=O), 1475.16 cm^−1^ (C=C), and 1134.25 cm^−1^ (C–N). After nanoparticle formation, these peaks shifted to 3502.47, 2922.60, 1636.87, 1388.61, and 1034.79 cm^−1^.

The observed shifts in the CTAB system primarily result from electrostatic interactions between CTAB's quaternary ammonium groups and charged functional groups of neomycin, unlike the dominant hydrogen bonding mechanism in the PVP system. Comparison of these results with previous studies^29,59,66^ demonstrates that both stabilizer systems effectively maintain neomycin's structural integrity during nanoparticle formation, yet employ distinct crystallization pathways. The PVP system, through its lattice of hydrogen bonds, produces nanoparticles with size distribution characteristics different from CTAB, while the CTAB system generates nanoparticles with superior morphology in this study.

These differences in stabilization mechanisms directly impact the final physicochemical properties of the nanoparticles, including colloidal stability, drug release behavior, and pharmaceutical activity of the nanoparticles. These results not only compare and analyze the different stabilization mechanisms of PVP and CTAB stabilizers, but also establish a valuable foundation for selecting appropriate stabilizers based on the intended application of neomycin nanoparticles or other pharmaceutical nanoparticles.^29,59,66^

### 3.2. Effect of agitation rate on induction time and particle size

**Fig. 13:**
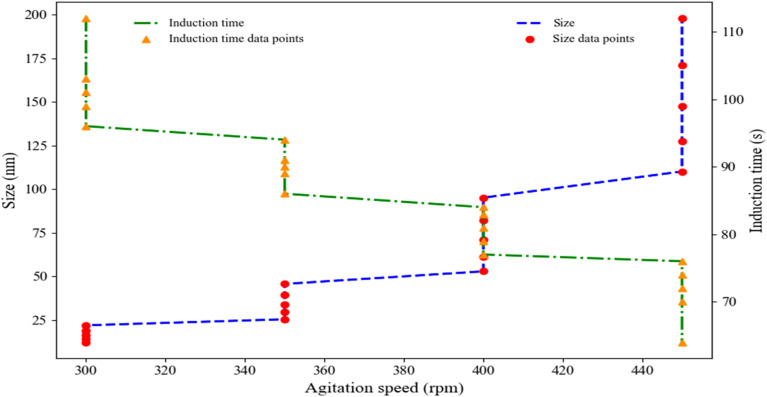
Effect of agitation rate on induction time and size of neomycin nanoparticles in the presence CTAB.

The agitation rate is a key parameter that significantly affects the quality and kinetics of nanoparticle synthesis, including crystallization, supersaturation, and crystal growth.^67^ Increasing the agitation rate improves the diffusion coefficient and reduces mass transfer resistance, thereby shortening the induction time.^68^ In this study, the effect of agitation rate on the induction time and particle size of neomycin nanoparticles in the presence of CTAB stabilizer was investigated and their presented in Fig. 13.^29,69^ Experiments were conducted at a constant temperature of 25 °C using four different agitation rates (300, 350, 400, and 450 rpm). The results demonstrated an inverse relationship between agitation rate and induction time, with higher rates causing a significant reduction in induction period. Furthermore, increased agitation rates reduced supersaturation levels, preferentially promoting particle growth over nucleation and ultimately leading to larger average nanoparticle sizes.^70^

From a mechanical perspective and in terms of process intensification, higher stirring rates enhance turbulence and increase diffusion coefficients. This leads to process intensification and stronger interactions between neomycin (solute), water (solvent), and the antisolvent (acetone). Consequently, by accelerating mixing, it reduces supersaturation.^71^ These conditions decrease mass transfer resistance while delaying the adsorption of CTAB stabilizer molecules onto neomycin crystal surfaces. This mechanism helps maintain optimal supersaturation ratios while minimizing induction time. Based on the experimental findings, distinct stirring rates were selected for each set of five replicate trials. This approach was adopted to establish an optimal balance among three critical parameters: stabilizer diffusion, induction time control, and supersaturation management. This would result in the production of neomycin nanoparticles with polydisperse size distribution and high stability.

### 3.3. Solubility measurement of neomycin

**Fig. 14:**
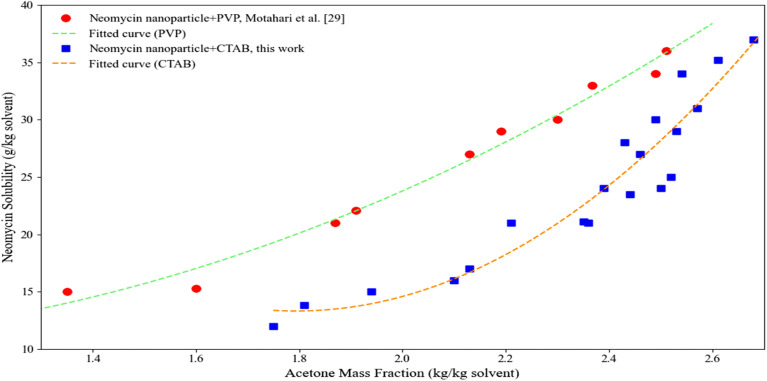
Neomycin solubility *versus* acetone mass fraction in the presence of CTAB and comparison with existing results.^29^

This study investigated the solubility enhancement of neomycin in the presence of CTAB stabilizer through controlled dynamic shock application. In contrast to the reference study,^29^ which utilized PVP at a mixing speed of 300 rpm, the present experimental design involved the addition of 0.3 g neomycin to a 10 g solvent–antisolvent mixture under 350 rpm agitation, incorporating intermittent dynamic shocks consisting of 3-second pulses at 380 rpm. This optimized hydrodynamic regime created controlled turbulence that effectively reduced boundary layer thickness while preventing amorphous impurity formation.^72^ The results demonstrated that CTAB-containing systems achieved acetone mass fractions of 1.75–2.68, representing an 18–22% solubility enhancement compared to PVP-based systems (1.35–2.51) (see Fig. 14). This significant improvement stems from synergistic effects between: (1) CTAB's molecular interactions (ionic complexation with neomycin molecules, surface tension reduction, and stable colloidal environment through spherical micelle formation) and (2) dynamic shock-induced phenomena (enhanced antisolvent penetration and uniform stabilizer distribution).^73^ Comprehensive analysis revealed that this combined approach not only increases saturation solubility but also promotes bimodal size distribution by preventing nanoparticle aggregation. The findings underscore the critical importance of concurrent optimization of physicochemical parameters in antisolvent crystallization processes for pharmaceutical nanoparticles.^74^

### 3.4. Determination of induction time, supersaturation, solubility and nucleation mechanism in the presence of CTAB

This study comprehensively investigated the influence of CTAB surfactant on neomycin crystallization. According to the experimental results and DLS data analysis, the presence of CTAB at 0.6 g kg^−1^ concentration significantly modifies key crystallization parameters. The supersaturation (S), as the primary driving force, directly affects the induction time, critical radius, growth rate, solubility, nanoparticle size, and nucleation mechanism.^33^ CTAB operates through selective adsorption on crystalline surfaces, substantially influencing the nucleation pathway.^75,76^ Simultaneously, steric-electrostatic stabilization by CTAB enhances colloidal stability (maintained for several days) and prevents particle agglomeration. These results, consistent with previous studies,^29,33,77^ demonstrate that CTAB's dual mechanism modulating nucleation while controlling growth enables the production of uniform nanoparticles with optimal size distribution and morphology.^78^ Quantitative data for these parameters are presented in Table 2, with the growth rate (intensity %) and nanoparticle size data being experimentally derived from DLS analysis.

**Table 2:** Solubility, induction time, critical radius and supersaturation concentration at the nucleation point of neomycin sulfate in the presence of CTAB solution

Agitation rate (rpm)	Neomycin initial concentration (g kg^−1^)	Solubility (g kg^−1^)	Supersaturation (S)	Induction time (s)	Critical radius (nm)
300	20	17	1.18	112	1.689
	20	16	1.21	103	1.467
	20	15	1.33	91	0.981
	20	13.8	1.45	86	0.753
	20	12	1.66	79	0.552
350	30	25	1.2	101	1.534
	30	24	1.25	94	1.254
	30	23.5	1.28	89	1.133
	30	21.1	1.43	81	0.782
	30	21	1.67	74	0.545
400	40	31	1.29	99	1.099
	40	29	1.379	96	0.957
	40	27	1.48	83	0.714
	40	24	1.66	76	0.552
	40	21	1.9	72	0.436
450	50	37	1.351	90	0.932
	50	35.2	1.408	84	0.817
	50	34	1.47	77	0.726
	50	30	1.666	70	0.548
	50	28	1.785	64	0.483

By plotting ln(*t*_ind_) *versus* ln(*S*) and ln(*t*_ind_) *versus* 1/(ln(*S*))^2^, the nucleation mechanism can be identified. According to eqn S3, if the relationship between ln(*t*_ind_) and 1/(ln(*S*))^2^ is linear, then the nucleation mechanism aligns with the Classical Homogeneous Nucleation Model. Conversely, if the relationship between ln(*t*_ind_) and ln(*S*) is linear, secondary nucleation is recognized as the dominant mechanism (as described in eqn S5). A comprehensive theoretical discussion and the derivations of the equations (and equation numbers) are provided in the SI.

**Fig. 15:**
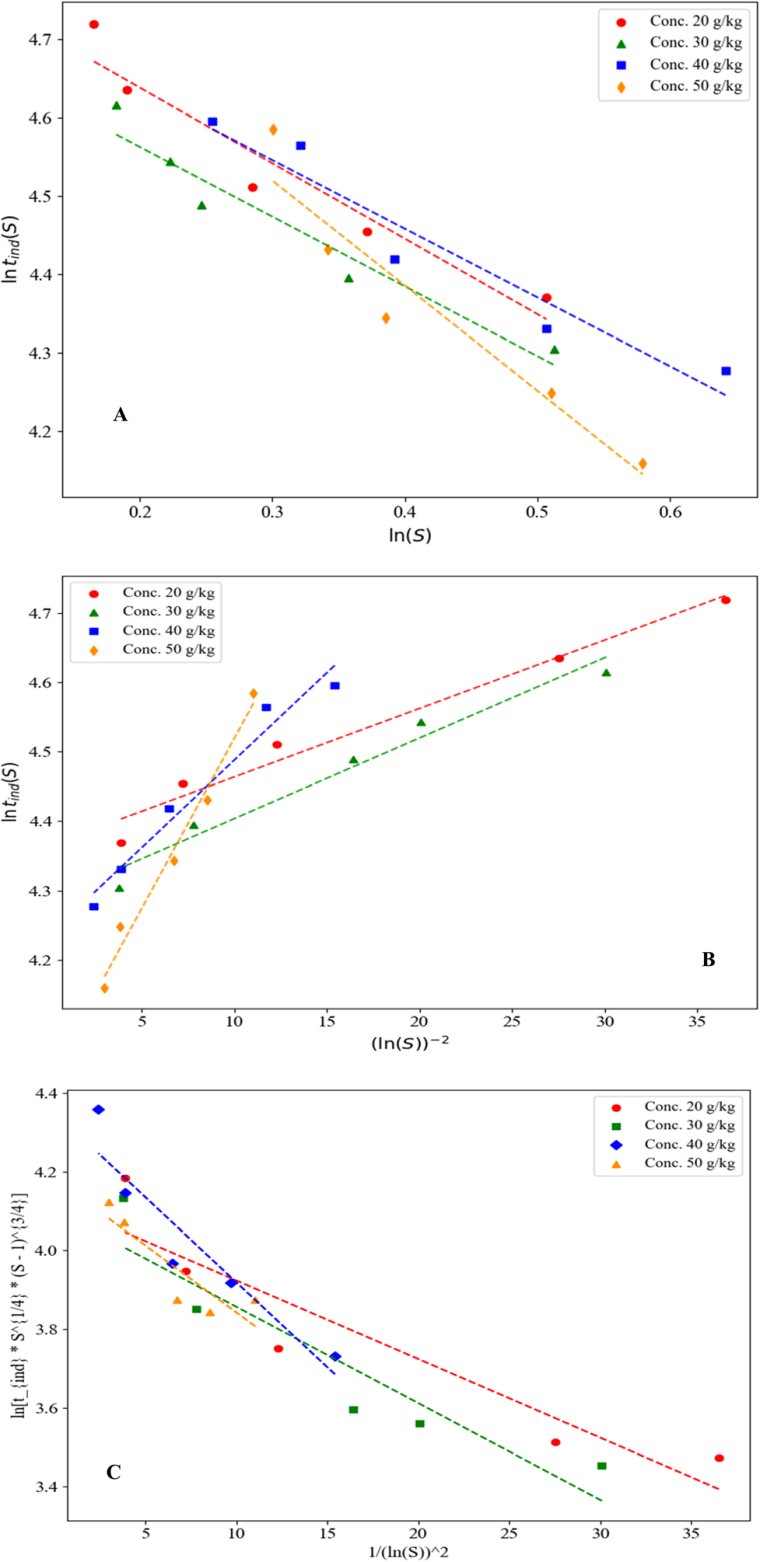
(A) Ln *t*_ind._*v.s* 1/(ln *S*)^2^ and (B) ln *t*_ind._*v.s* ln *S* and (C) ln(*t*_ind._*S*^(1/4)(*S*−1)^(3/4)^) *versus* 1/(ln(*S*))^2^ at temperatures of 25 °C and the initial concentration of 0.6 g kg^−1^ of CTAB.

Furthermore, by plotting ln(*t*_ind._*S*^(1/4)(*S*−1)(3/4)^) against 1/(ln(*S*))^2^, the nucleation mechanism can be analyzed using Kashchiev's heterogeneous nucleation model (eqn S10, with *m* = 1). In this regard, the plots of ln(*t*_ind_) *versus* 1/(ln(*S*))^2^, ln(*S*), and ln(*t*_ind._*S*^(1/4)(*S*−1)(3/4)^) *versus* 1/(ln(*S*))^2^ at 25 °C for initial concentrations of 20, 30, 40, and 50 g kg^−1^ of neomycin sulfate, as well as an initial concentration of 0.6 g kg^−1^ of CTAB, have been illustrated in Fig. 15a–c.

The linear regression analysis of all three plots (*R*^2^ values presented in Table 3 for Fig. 15a–c) reveals that homogeneous primary nucleation, as described by classical nucleation theory, shows considerably higher correlation coefficients compared to secondary nucleation mechanisms when evaluated using both classical nucleation theory and Kashchiev heterogeneous nucleation model (*m* = 1) at an initial CTAB concentration of 0.6 g kg^−1^. The experimental conditions strongly support these findings, as the crystallization process was conducted without any seeding technique, with nanoparticles forming spontaneously from a highly supersaturated solution that exceeded the MSZW.^79^ The excellent agreement between the theoretical models and experimental results (including critical radius measurements, growth rate data, and final particle size distribution) confirms the dominance of homogeneous primary nucleation in this system.^80^ Furthermore, as shown in Fig. 1D, the achieved supersaturation levels placed the system well into the labile zone, consistent with the theoretical frameworks of Mullin ^15^ and Myerson,^16^ providing additional evidence that homogeneous primary nucleation prevails over secondary mechanisms under these experimental conditions. Considering that, for the Kashchiev heterogeneous nucleation model, the results for *m* = 1 provided better agreement compared to *m* = 0.5 and *m* = 0.33, this study uses *m* = 1.

**Table 3:** Correlation coefficients of different nucleation models in the presence of 0.6 g kg^−1^ CTAB solution

Initial neomycin concentration (g Kg^−1^)	Line equation, *R*^2^		
	Secondary nucleation model	Classical homogeneous nucleation model	Kashchiev heterogeneous nucleation model
20	−0.96*X* + 4.83; 0.9297	0.01*X* + 4.36; 0.9728	−0.04*X* + 4.35; 0.8702
30	−0.89*X* + 4.74; 0.9406	0.01*X* + 4.29; 0.9647	−0.03*X* + 4.18; 0.8662
40	−0.88*X* + 4.81; 0.9275	0.03*X* + 4.24; 0.9653	−0.02*X* + 4.12; 0.8815
50	−1.34*X* + 4.92; 0.9120	0.05*X* + 4.03; 0.9818	−0.02*X* + 4.10; 0.7703

**Fig. 16:**
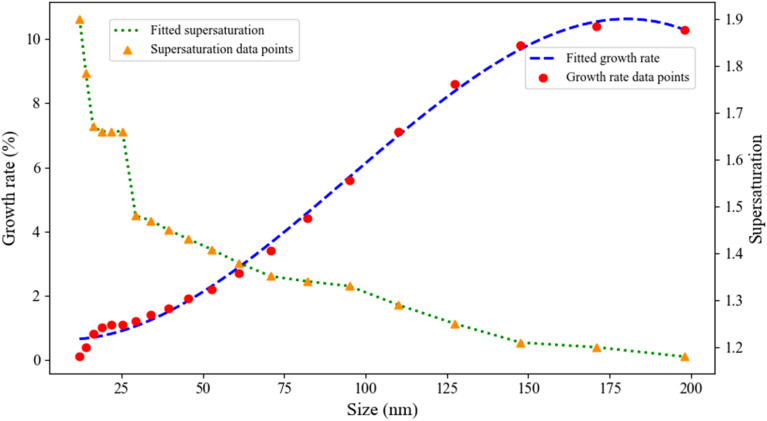
Direct effect of supersaturation on the growth rate and size of neomycin nanoparticles in the presence of CTAB.

The experimental study of the relationships between supersaturation, growth rate, and nanoparticle size at 25 °C (Fig. 16) reveals a distinct inverse correlation between supersaturation level and growth kinetics during neomycin nanoparticle formation.^81^ As observed, decreasing supersaturation levels lead to increased growth rates, ultimately producing larger nanocrystals a phenomenon consistent with mass-transfer-limited kinetic models describing nucleation-growth equilibrium. This behavior persists until reaching critical particle dimensions, where growth rate attenuation occurs due to: diffusion-dependent mass transfer limitations, and stabilizing effects of CTAB through surface adsorption that effectively suppresses particle aggregation.^40,73,82^ The cationic surfactant CTAB facilitates polydisperse particle distribution by forming structured cationic bilayers on crystal surfaces, which simultaneously provide colloidal stability and modulate growth kinetics.^83^ Obtained results demonstrate that precise coordination of three critical parameters supersaturation control, growth rate optimization, and optimal surfactant concentration effectively controls final nanoparticle dimensions within the desired range.^84^ The proposed growth kinetic model incorporates both classical crystallization theory and nanoscale surface stabilization effects, showing excellent agreement with experimental data across all studied parameters and DLS/size distribution analysis results.^85^

**Fig. 17:**
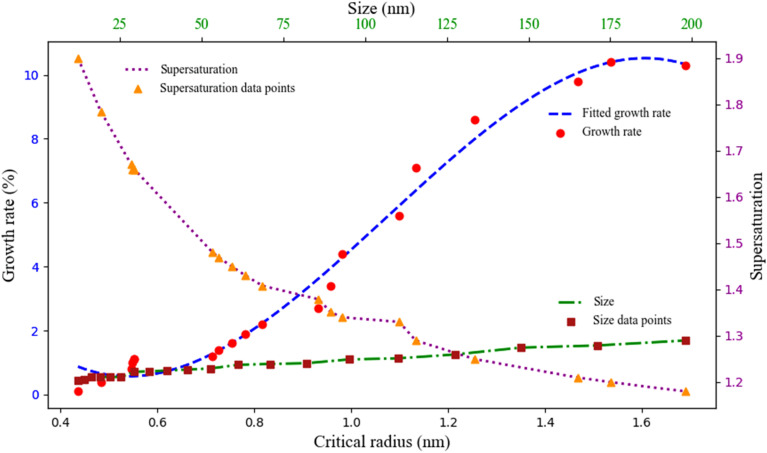
Direct relationship between critical radius and supersaturation and its effect on the growth rate and size of neomycin nanoparticles in the presence of CTAB.

As previously explored in relation to supersaturation, growth rate, and nanoparticle size (Fig. 16), the experimental results shown in Fig. 17 further illustrate the critical radius, growth kinetics, and nanoparticle size across varying supersaturation levels.^86^ Based on data obtained from DLS analysis and critical radius calculations (eqn S16), it is observed that decreasing supersaturation leads to a significant increase in crystal growth rates, resulting in the formation of larger nanoparticles.^14–16,87^ Conversely, increasing supersaturation levels significantly accelerate primary nucleation processes (which will be discussed in detail in subsequent figures) while simultaneously causing a noticeable reduction in crystal growth rates.^88^ This phenomenon follows fundamental crystallization kinetics principles where, at high supersaturation levels, the elevated concentration of solute species leads to the formation of a greater number of primary nuclei.^89^ Simultaneously, the distribution of solute molecules among these numerous nuclei results in decreased growth rates for crystals, ultimately producing smaller nanoparticles with a bimodal size distribution.^86^ The role of the CTAB stabilizer in this system is particularly crucial. Through surface adsorption mechanisms, CTAB not only prevents nanoparticle aggregation but also precisely modulates growth kinetics, enabling better control over final particle dimensions.^90^ These synergistic effects contribute to the formation of colloidally stable nanoparticles with uniform size distributions. An important observation is the direct correlation between nucleation duration and critical particle size, where extended nucleation times cause the formation of smaller particles.^91^ Furthermore, dynamic parameters such as agitation rate and controlled shock pulses serve as effective tools for fine-tuning nanoparticle characteristics.^92^

**Fig. 18:**
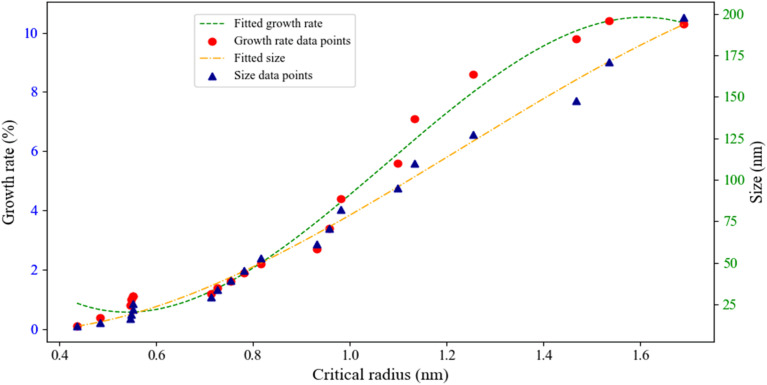
Effect of critical radius on the growth rate and final size of neomycin nanoparticles in the presence of CTAB stabilizer.

In conventional crystallization processes, nucleation is typically avoided as the primary objective is to produce larger-sized particles.^93^ However, in pharmaceutical nanoparticle production, achieving particles with controlled size and morphology requires molecular stabilization and directing the system toward nucleation mechanisms rather than crystal growth.^94^ As demonstrated by Motahari *et al.*,^29^ this can be accomplished by exceeding the metastable zone and creating conditions of high supersaturation, which increases nucleation rate while inhibiting particle growth.^95^ In this study, controlled supersaturation conditions were established through gradual addition of acetone (as antisolvent) to the ternary water-neomycin-CTAB system.^96^ As shown in Fig. 1 (parts A–D), physical changes in the solution occur when neomycin concentration exceeds its solubility limit, leading to primary nucleus formation. Due to the inherent instability of supersaturated conditions, the system tends to eliminate excess neomycin until reaching equilibrium solubility concentration.^97^ The stability of these nuclei critically depends on the critical radius (*r*_c_), where particles larger than *r*_c_ remain stable while smaller nuclei tend to redissolve.^15,16^ CTAB plays a crucial stabilizing role through multiple mechanisms: reducing surface energy and increasing critical radius, providing colloidal stability *via* steric-electrostatic stabilization, and simultaneously controlling growth rate and final nanoparticle size. Results demonstrate in Fig. 18, that enhanced nanoparticle stability and increased critical radius lead to greater particle growth and final size. However, exceeding the optimal CTAB concentration (0.6 g kg^−1^) induces micelle formation, diminishing its stabilizing effectiveness.^98,99^

### 3.5. Interfacial energy

**Fig. 19:**
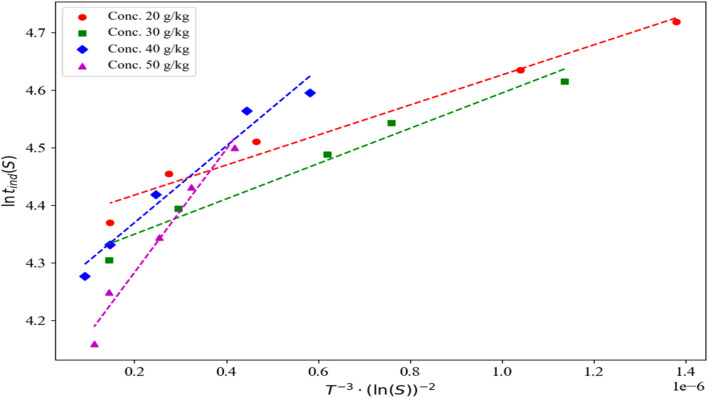
Ln *t*_ind_*v.s* 1/(*T*^3^ (ln *S*)^2^) for CTAB solution concentrations of 0.6 g kg^−1^ at 25 °C.

Interfacial energy at the solid–liquid interface, can typically be regarded as an intrinsic property of the system.^100^ It is indeed a key parameter strongly influenced by the solvent composition and the presence of surfactants such as CTAB.^29,101^ In this study, the effect of CTAB at a constant concentration of 0.6 g kg^−1^ on the interfacial energy of the crystallization system of neomycin at various concentrations (20, 30, 40, and 50 g kg^−1^) was investigated at a temperature of 25 °C. Key system parameters included the density of neomycin (*ρ* = 640 kg m^−3^), measured using the volume displacement method; the shape factor (*f* = 0.058)^16,29^ and the molar mass (*M* = 908.88 g mol^−1^). In order to calculate the interfacial energy, based on the theoretical framework, elaborated in Section 2.3, results obtained from analyzing the ln *t*_ind_*versus* 1/*T*^3^(ln *S*)^2^ plot in Fig. 19 indicated that increasing the mass fraction of acetone from 2.51 (ref. 29) to 2.68 kg kg^−1^ of the solvent led to a significant reduction in Interfacial energy. At various concentrations of neomycin (20, 30, 40, and 50 g kg^−1^), a comparison was made between the system stabilized with CTAB and previous studies that utilized PVP as a stabilizer.^29^ As shown in Table 4, the presence of CTAB resulted in distinctly different behavior in terms of nanoparticle kinetics and morphology. This distinction can be attributed to a significant reduction in surface energy in the presence of CTAB, as well as distinct molecular interactions between neomycin and the cationic surfactant, which subsequently influenced solubility and nucleation behavior.^102^ The increase in agitation rate, combined with the presence of CTAB, exhibited a notable synergistic effect on the kinetic stability of the system. Although comparative data for CTAB-stabilized systems are limited in previous literature, the current results demonstrate a remarkable reduction in surface energy and improved particle size uniformity compared to PVP-stabilized systems. The proposed mechanism for this observation includes the effective adsorption of CTAB onto nanoparticle surfaces, alterations in the local solubility of neomycin, and the formation of stable electrical double layers at the interface.^103^ Furthermore, increased agitation enhanced mass transfer, improved surfactant distribution, and promoted the formation of stable nuclei, ultimately leading to a significant reduction in surface energy and effective suppression of nanoparticle aggregation.^104^

**Table 4:** Comparison of interfacial energy for CTAB solution concentrations of 0.6 g kg^−1^ at 25 °C with existing results. ^29^

Initial neomycin concentration (g kg_solvent_^−1^)	*R* ^2^	Interfacial energy this work (mJ m^−2^)	Interfacial energy (mJ m^−2^), Motahari *et al.*^29^
20	0.9728	1.33	7.68
30	0.9647	1.41	8.056
40	0.9653	1.9	—
50	0.9726	2.14	—

### 3.6. Measurements of MSZW

**Fig. 20:**
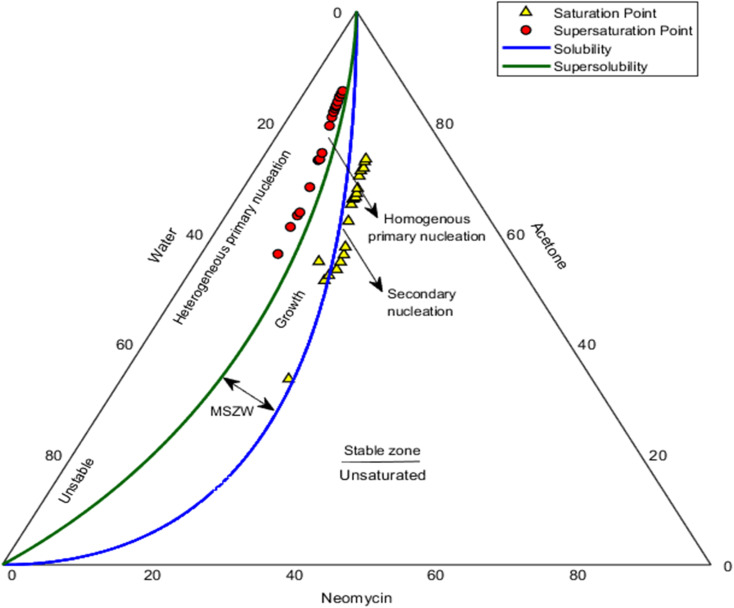
MSZW of neomycin as a function of weight percentage in the neomycin/water/acetone system in the presence of CTAB solution with concentrations of 0.6 g kg^−1^.

Studies show that the presence of additives significantly affects the thermodynamic and kinetic properties of nucleation, including changes in solubility, supersaturation, the MSZW, induction time, and nucleation rate.^105^ In this regard, the MSZW of neomycin as a function of weight percentage in the neomycin/water/acetone (solute/solvent/antosolvent) system in the presence of with CTAB as a stabilizer at a concentration of 0.6 g kg^−1^ and a temperature of 25 °C was investigated in the Fig. 20, (two phase nucleation).^29^

Saturation and supersaturation parameters are recognized as key factors in optimizing the crystallization product quality, directly influencing the purity, crystal size, and nucleation mechanisms.^106^ The maximum level of saturation and supersaturation tolerable by the system is directly associated with determining solubility and the metastable zone width, which in turn depends on several factors, including the type of solvent and antisolvent, the presence of stabilizers, impurities, seed crystals, stirring rate, and system temperature.^107,108^

In this research, using acetone as the antisolvent under controlled conditions (stirring rate and antisolvent addition rate, optimal CTAB concentration), three distinct regions stable, metastable, and unstable were identified. In the stable region with a low acetone concentration,^109^ no significant change in neomycin solubility was observed. With a gradual increase in acetone, the system entered the metastable region, where secondary nucleation and controlled crystal growth occurred. Finally, in the unstable region, homogeneous primary nucleation predominated, resulting in nanoparticles with uniform size distribution.^110^ A comparison of results with similar studies using PVP as a stabilizer^29^ revealed that although the system exhibited similar behavior within a specific range of neomycin concentrations, CTAB displayed distinctive performance at concentrations of 20–50 g kg^−1^. This distinction included a significant increase in the MSZW and enhanced colloidal stability of the nanoparticles, attributed to specific CTAB mechanisms such as electric double-layer formation, reduction of interfacial solid–liquid surface energy, and modulation of nucleation kinetics.^111^ The strong interaction between the quaternary ammonium groups of CTAB and the functional groups of neomycin led to selective adsorption and a reduction in the free energy of nucleation, shifting the critical solubility and supersaturation points in the system.^104^

Although several ternary diagrams have been developed to examine crystallization systems, interpreting these diagrams in the separation region can be challenging.^112^ Nevertheless, the findings of this study indicate that CTAB is a suitable choice for the production of neomycin nanoparticles due to its higher colloidal stability, better particle size control, improved nucleation yield, and enhanced predictability of system behavior.^113^ These results not only provide deeper insights into the crystallization mechanisms of pharmaceutical nanoparticles but also offer valuable practical strategies for designing more efficient industrial processes and producing nanoparticles with controlled physicochemical properties.

## 4. Conclusions

This study presents a comprehensive investigation into the synthesis process of neomycin nanoparticles through anti-solvent induced crystallization, utilizing CTAB surfactant. The results demonstrated that precise control over process parameters including induction time, supersaturation levels, agitation rate, and optimal CTAB concentration leads to the production of nanoparticles with bimodal size distribution and high colloidal stability. Induction time was measured *via* an online turbidity method, revealing that high supersaturation levels promote homogeneous primary nucleation mechanisms over other nucleation pathways. Moreover, elevated supersaturation significantly influences nucleation rate, critical radius, growth rate, and ultimately, the size of neomycin nanoparticles. CTAB effectively enhanced crystallization control by reducing surface energy and broadening the metastable zone width. Advanced characterization techniques such as SEM, TEM, XRD, FT-IR, TGA, and DSC confirmed the formation of crystalline nanoparticles with modified surface properties and improved thermal stability. Additionally, the dynamic shock technique employed in this research proved to be an effective tool for influencing induction time and supersaturation, thereby managing nucleation rate and particle growth.

## Conflicts of interest

There are no conflicts to declare.

## Supplementary Material

RA-015-D5RA08649C-s001

## Data Availability

The authors confirm that all relevant data supporting the findings of this study are included within the submitted manuscript. If any additional raw data files in alternative formats are needed, they can be made available upon reasonable request from the corresponding author. The source data accompanying this paper is also provided. Supplementary information is available. See DOI: https://doi.org/10.1039/d5ra08649c.
