# Supplementary material for: Impact of supersaturation on growth, critical radius, and size in neomycin nanoparticle crystallization using anti-solvent and CTAB
Source: RSC Adv. 2025 Dec 3;15(56):47766–89. doi: 10.1039/d5ra08649c (PMC12679610; doi:10.1039/d5ra08649c)
Supplement: RA-015-D5RA08649C-s001 [file RA-015-D5RA08649C-s001.pdf]

Supplementary Information (SI) for RSC Advances.
This journal is © The Royal Society of Chemistry 2025

Supporting Information for the manuscript of

"Impact of Supersaturation on Growth, Critical Radius, and Size in Neomycin Nanoparticle Crystallization Using Anti-Solvent and CTAB"

1. Materials and experimental procedure

1.1. Materials

The neomycin sulfate (99.8%) was purchased from Sigma-Aldrich. CTAB and acetone (CH3COCH3 , 99.9%) were provided from Merck.

1.2. Material characterization

The samples were comprehensively characterized using a suite of analytical techniques. Morphology and elemental composition were investigated using ZEISS instruments: field emission scanning electron microscopy (FESEM, Sigma VP model) equipped with energy-dispersive X-ray spectroscopy (EDX), and transmission electron microscopy (TEM, EM10C-100KV model). Particle size distribution was analyzed by dynamic light scattering (DLS) using a Malvern Zetasizer. Thermal properties were assessed using Mettler Toledo equipment: thermogravimetric analysis and derivative thermogravimetry (TGA/DTG, TGA2 model) for stability, and differential scanning calorimetry (DSC, DSC-2 model) for thermal transitions. The crystalline structure was determined by X-ray diffraction (XRD, Panalytical X'Pert Pro model), and functional groups were identified by Fourier-transform infrared spectroscopy (FT-IR, PerkinElmer Spectrum Two model). Surface topography was evaluated by atomic force microscopy (AFM, Bruker JPK Nanowizard-2 model). And also Mapping and EDX-a was done by Vega 3 model aof TESCAN Company.

1.3. Preparation of CTAB solution

To prepare the CTAB solution, 0.365 g of CTAB powder (molecular weight = 364.45 g/mol) was dissolved in 1.0 liter of double-distilled water and stirred thoroughly to achieve an aqueous solution with a concentration of 1.00151 mM. The addition of CTAB is hypothesized to reduce

the surface tension of the formed nanoparticles, a phenomenon attributed to its significant surfactant properties [39, 40]. This solution was stored at room temperature for five days and stirred twice daily (morning and evening) to ensure homogeneity and stability. The final CTAB solution was subsequently utilized as a stabilizing agent in the synthesis of neomycin nanoparticles.

1.4. Synthesis of neomycin nanoparticles

In this section, the method for the synthesis of neomycin nanoparticles is explained in detail. First, an exact amount of neomycin powder (purity ≥ 95%) was weighed using a digital analytical balance (accuracy: ±0.1 g) based on the desired equilibrium concentration. The weighed neomycin was then added to 10 g of double-distilled water (25°C). To ensure complete dissolution, the mixture was homogenized using a magnetic stirrer at controlled speeds of 300, 350, 400, and 450 rpm for 20 minutes. This range of stirring speeds was selected to investigate the effect of shear force on solution homogeneity. After confirming the homogeneity of the neomycin solution, a predetermined amount of CTAB surfactant with a concentration established from prior studies was added to the beaker. To achieve optimal mixing, the stirring system was activated at a constant speed between 300 and 450 rpm, and the mixing process continued until equilibrium was reached. The solution temperature was continuously monitored using a calibrated digital thermometer (±0.1°C). A turbidity sensor was installed 2 cm from the crystallizer wall to measure changes in turbidity. Pure acetone (HPLC grade) was added dropwise (0.02 mL per drop) at 5-minute intervals. The first signs of physical change (e.g., localized cloudiness) were recorded as the initial saturation point. Acetone addition continued until stable turbidity (supersaturation) was achieved under isothermal conditions (25°C). The induction time (the interval between initial saturation and supersaturation), as clearly depicted in Fig.1, Section C (the induction time segment of steps 1 to 4), was measure using a synchronized turbidimeter and reaction timer data. To ensure reproducibility, the entire process was repeated in four independent trials under controlled conditions (constant temperature, humidity, and lighting). The schematic diagram of the used laboratory setup is shown in Fig.2.

It should be noted that, to mitigate the totoxicity of CTAB, the surfactant was extensively removed post-synthesis. The crude nanoparticle suspension was subjected to centrifugation (12,000 rpm, 30 min, 4 °C) to eliminate free CTAB and acetone. The precipitate was then rigorously washed via

five cycles of redispersion and centrifugation in a cold ethanol-water solution (70:30 v/v, 4 °C) to disrupt CTAB's hydrophobic interactions, followed by a final wash with cold deionized water. Efficacy of CTAB removal was quantitatively confirmed by EDS analysis, which showed a 96% reduction in bromine content (a signature element of CTAB) from 48.5% (Fig. 6a, before washing) to 2% ((Fig. 6b, after washing) after the washing process.

2. Theory

2.1. Classical nucleation model

Classical nucleation theory offers a theoretical basis for the formation of new phases such as solid, liquid, or gas within an existing parent phase during phase transitions [1]. This framework explains how tiny clusters, or nuclei, of a new phase originate and expand under specific conditions. A key aspect of this theory is the steady-state nucleation rate, which quantifies the number of nuclei generated per unit time and volume. This rate is commonly expressed using an Arrhenius-type equation:

$$J_s = Aexp(-\frac{\Delta G_{crit}}{kT}) \tag{1}$$

Here, A represents the pre-exponential factor, which is influenced by the nucleation kinetics within the growth medium. The parameters k and T correspond to Boltzmann's constant and the absolute temperature (K), respectively. Additionally, the correlation between temperature and the supersaturation ratio (S) is mathematically defined in Eq. 2.

$$J_s = \text{Aexp}\left[-\frac{16\,\pi\gamma^3 v^2}{3k^3T^3(\text{Ln}\,S)^2}\right] \tag{2}$$

Given that the nucleation rate is inversely related to the induction time it can be said that the induction time in the homogeneous primary nucleation mechanism can be expressed in the following form [15, 29, 41]:

$$t_{ind} = A_1 exp\left[\frac{16\,\pi\gamma^3 v^2}{3k^3T^3(\text{Ln}\,S)^2}\right] \tag{3}$$

in which A_1 is the nucleation constant, γ is the solid–liquid surface tension and v is the molecular volume.

It should also be noted that the classical power equation presented in Eq. 4 has been used as a proposed model for secondary nucleation in the absence and presence of a solid phase:

$$J = K_s S^n \quad (4)$$

In this relation, K_s is an empirical constant related to the rate of secondary nucleation and n represents the order of secondary nucleation [2, 3].

The induction time for secondary nucleation in the absence of seed crystals can be expressed as Eq. 5.

$$\ln t_{ind} = \ln K_s - n \ln S \quad (5)$$

It is worth mentioning that in Eq. 5 by plotting ($\ln t_{ind}$) against (ln S) and calculating the slope of the resulting line, the degree of secondary nucleation (n) can be determined. This approach enables the analysis of nucleation behavior and contributes to a deeper understanding of the mechanisms governing the crystallization process.

2.2. Kashchiev heterogeneous nucleation model

In this section, the model proposed by Kashchiev et al. [4, 5], for calculating the induction time in the heterogeneous primary nucleation mechanism is presented.

$$t_{ind} = K exp\left(-\frac{S}{kT}\right)\left(1 - \exp\left(-\frac{S}{kT}\right)\right)^{\left(-\frac{3m}{1+m}\right)} . exp\left(\frac{4c^3 v^{23}}{27(1+3m)kTS^2}\right) \quad (6)$$

In which c is shape factor, and m is related to growth type.

The relationship in Eq. 6 can be summarized as follow:

$$t_{ind} = K\left(S(S-1)^{3m}\right)^{\frac{-1}{1+3m}} . exp\left(\frac{\gamma}{(1+3m)ln^2 S}\right) \quad (7)$$

Kashchiev et al. [4] has reported values of 1, 0.5, and 0.33 for the m parameter. The selection of any of these three values for the proposed model is made by fitting the experimental data to Eq. 7. In this article, the value of m is considered to be equal to 1; therefore, Eq. 7 can be expressed as follows:

$$t_{ind} = K\left(S(S-1)^3\right)^{\frac{-1}{4}} . exp\left(\frac{\gamma}{4ln^2 S}\right) \quad (8)$$

If the equation is written as follows:

$$t_{ind} . \left(S^{\frac{1}{4}}(S-1)^{\frac{3}{4}}\right) = K exp\left(\frac{\gamma}{4ln^2 S}\right) \quad (9)$$

If one takes the natural logarithm from Eq. 9 then:

$$ln(t_{ind}.S^{\frac{1}{4}}(S-1)^{\frac{3}{4}}) = \ln K + \frac{\gamma}{4ln^2S} \qquad (10)$$

Therefore, by plotting $ln(t_{ind}.S^{\frac{1}{4}}(S-1)^{\frac{3}{4}})$ against $(\frac{1}{ln^2S})$ and fitting the experimental data, the optimal value for m can be selected.

2.3. Interfacial energy calculations

The classical nucleation theory can be adapted for a solid-liquid system to determine interfacial energy, as expressed in Eq. 11 [6-8]:

$$J_s = \text{Aexp}\left[-\frac{f\gamma^3v^2}{k^3T^3(\text{Ln } S)^2}\right] \qquad (11)$$

In which f and γ are the particle shape factor and the interfacial energy of solid–liquid phase (J/m^2). The remaining parameters are consistent with those in Eq. 3. Given the inverse relationship between nucleation rate and induction time, Eq. 11 can be reformulated as Eq.12:

$$t_{ind} = A_1exp\left[\frac{f\gamma^3v^2}{k^3T^3(\text{Ln } S)^2}\right] \qquad (12)$$

If one takes the natural logarithm from Eq. 12 then:

$$\text{Ln } t_{ind} = \text{Ln } A_1 + \left[\frac{f\gamma^3v^2}{k^3T^3(\text{Ln } S)^2}\right] \qquad (13)$$

Therefore, by plotting Ln t_{ind} against $1/(T^3(\text{Ln S})^2)$ and finding the slope of the line (A), interfacial energy can calculated:

$$A = \frac{f\gamma^3v^2}{k^3} \qquad (14)$$

Finally, the interfacial energy can be calculated as Eq.15:

$$\gamma = k(\frac{A}{fv^2})^{1/3} \qquad (15)$$

In this equation v, represents the molecular volume (m^3) and is defined as v_m=M/ρN. Here ρ is density measured (kg/m^3), Mw indicates molecular weight (kg/mol), k is the Boltzmann constant, which has a value of 1.38×10^{-23} ($m^2\cdot kg/(s^2\cdot K)$), and N refers to Avogadro's number, equal to 6.022×10^{23} mol^{-1}.

It should be note that Eq. 16 can be used to calculate the critical radius [1].

$$r_c = \frac{2\gamma v}{kTln\,S} \qquad (16)$$

3. Figures of Manuscript

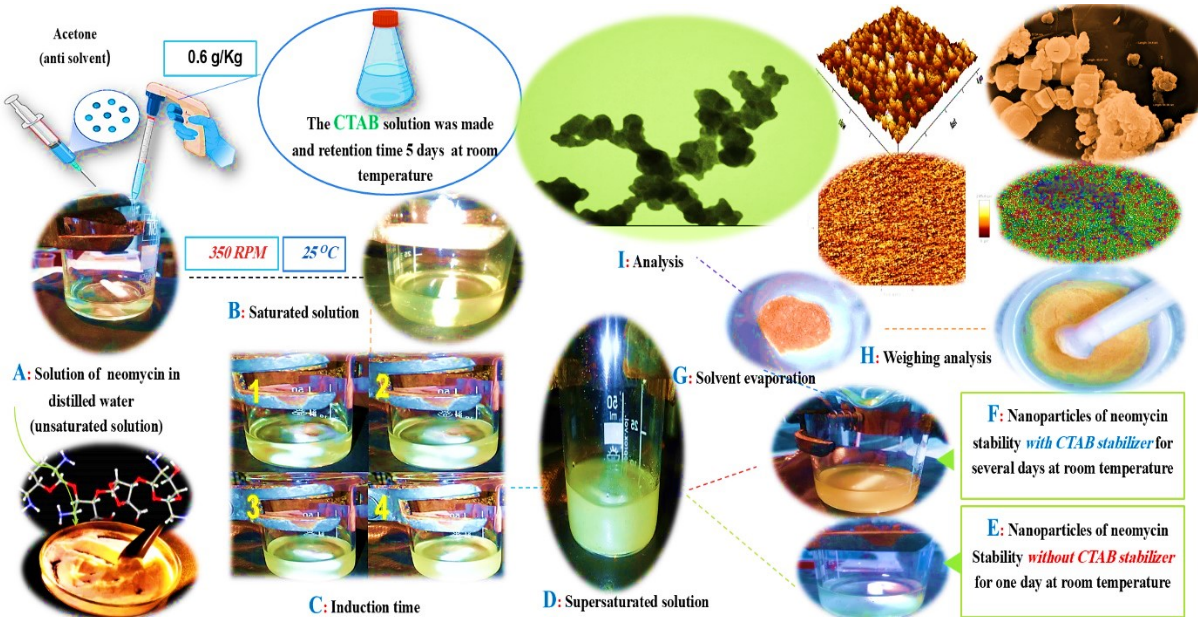

Fig. 1. The solution preparation to final results would include several consecutive stages: (A) unsaturated solution, (B) saturation state, (C) induction time measurement, (D) final supersaturation, (E) stability without CTAB, (F) stability with CTAB, (G) solvent evaporation, (H) weighing, and (I) nanoparticle characterization.

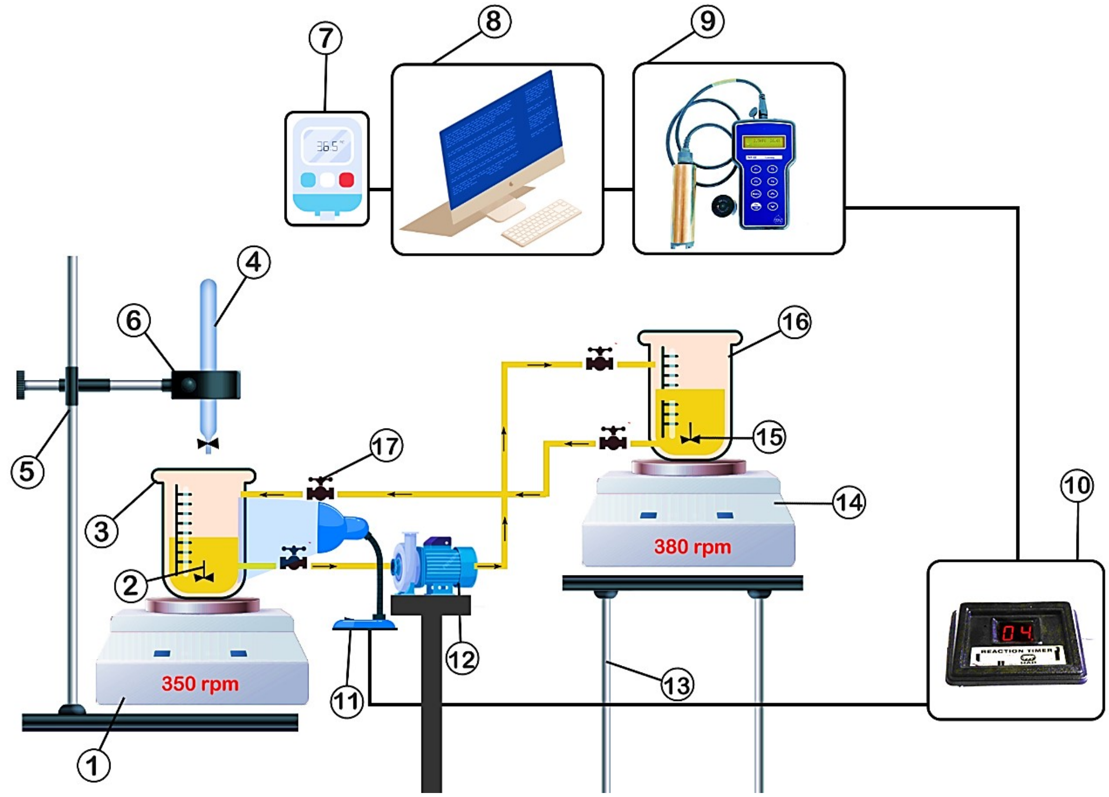

Fig 2. Schematic diagram of the laboratory setup: (1) First Stirrer, (2) Mixer or (Magnetic Stirrer), (3) First Crystallizer, (4) Burt, (5) Base, (6) Laboratory clamp, (7) Thermometer, (8) Pc Lab, (9) In situ turbidity meter, (10) Reaction timer and (11) LED, (12) Pump, (13) Support, (14) Second Stirrer, (15) Mixer or (Magnetic Stirrer), (16) Second Crystallizer and (17) Valve.

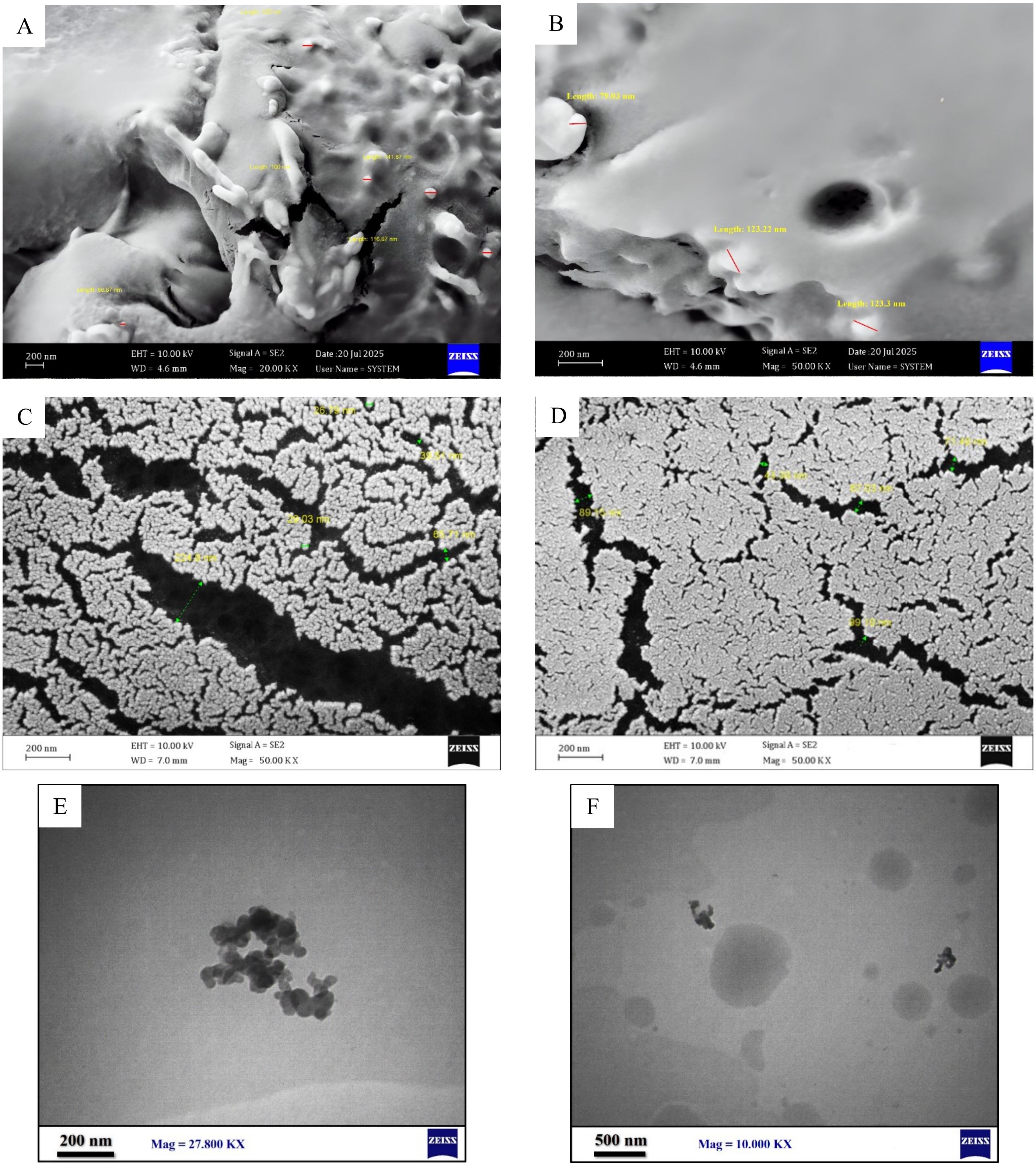

Fig. 3 A, B) FSEM (20.00 KX), C, D) FSEM (50.00 KX), E) TEM (27.800 KX) F) TEM (10.00 KX) and G) TEM (27.800 KX) images of nanoparticles without CTAB as stabilizer.

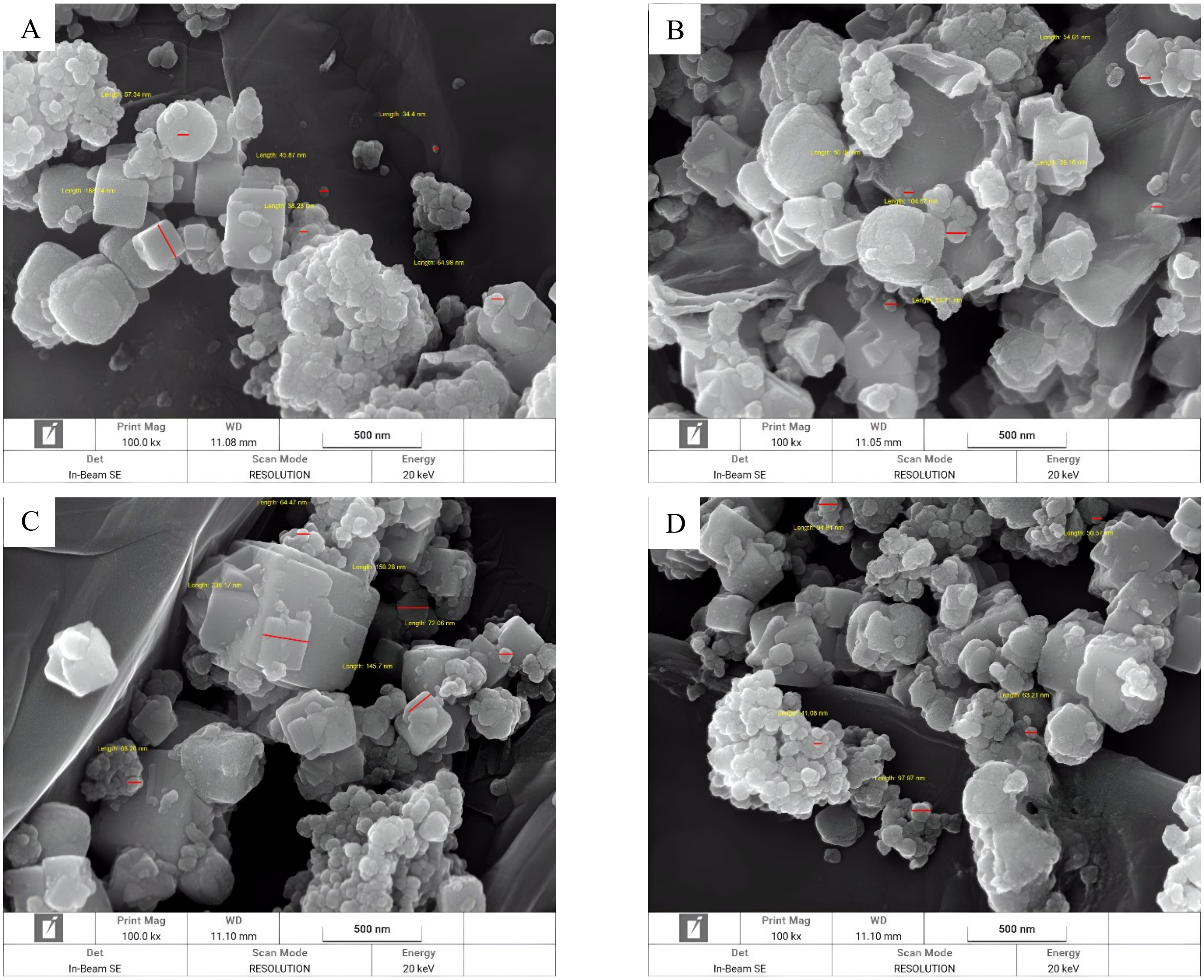

Fig. 4 FSEM images of synthesized neomycin nanoparticles (A-C) 100.00KX.

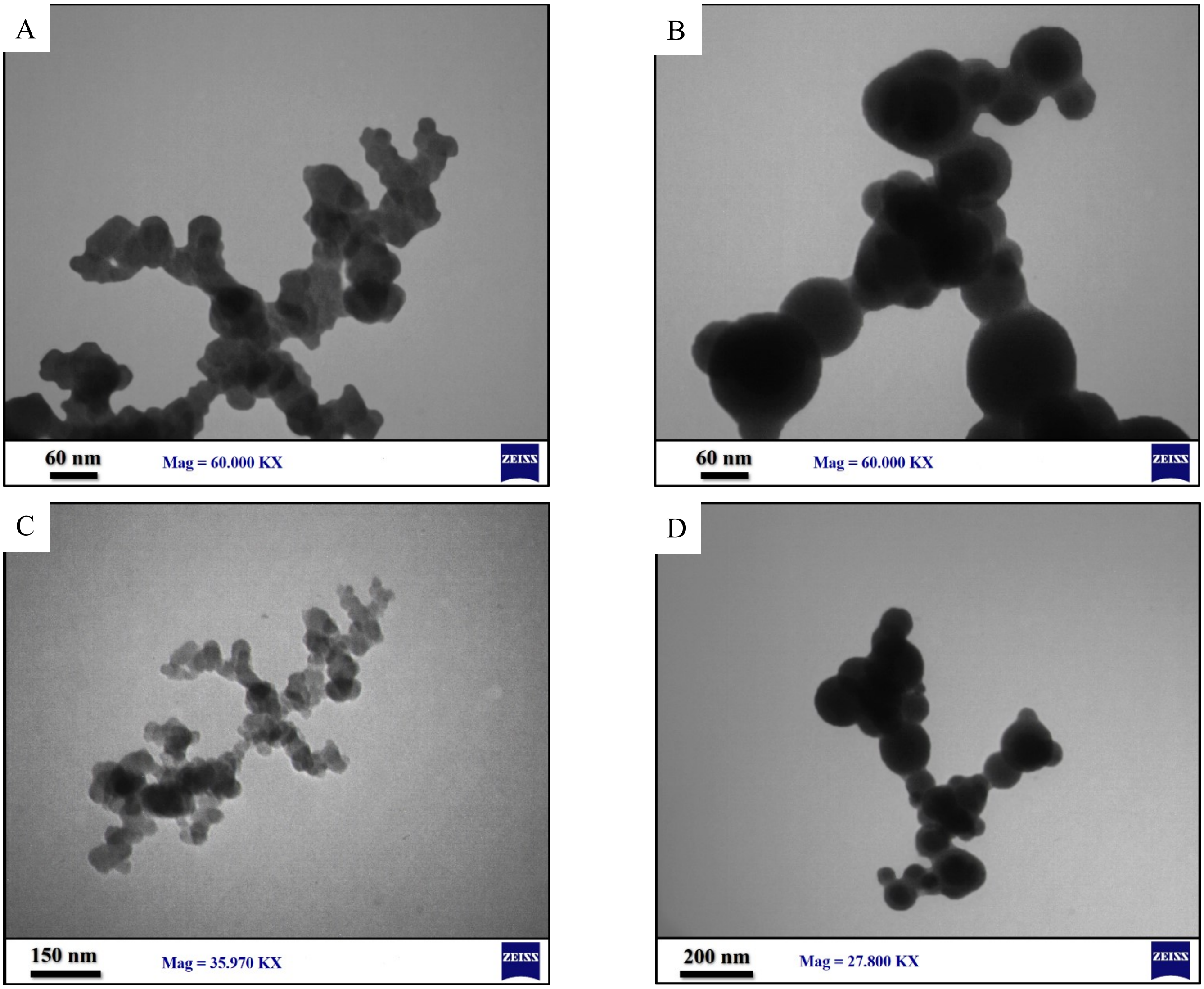

Fig. 5 TEM imaging of synthesized neomycin nanoparticles (A, B) 60.00 KX , C) 35.790 KX and D) 27.800 KX.

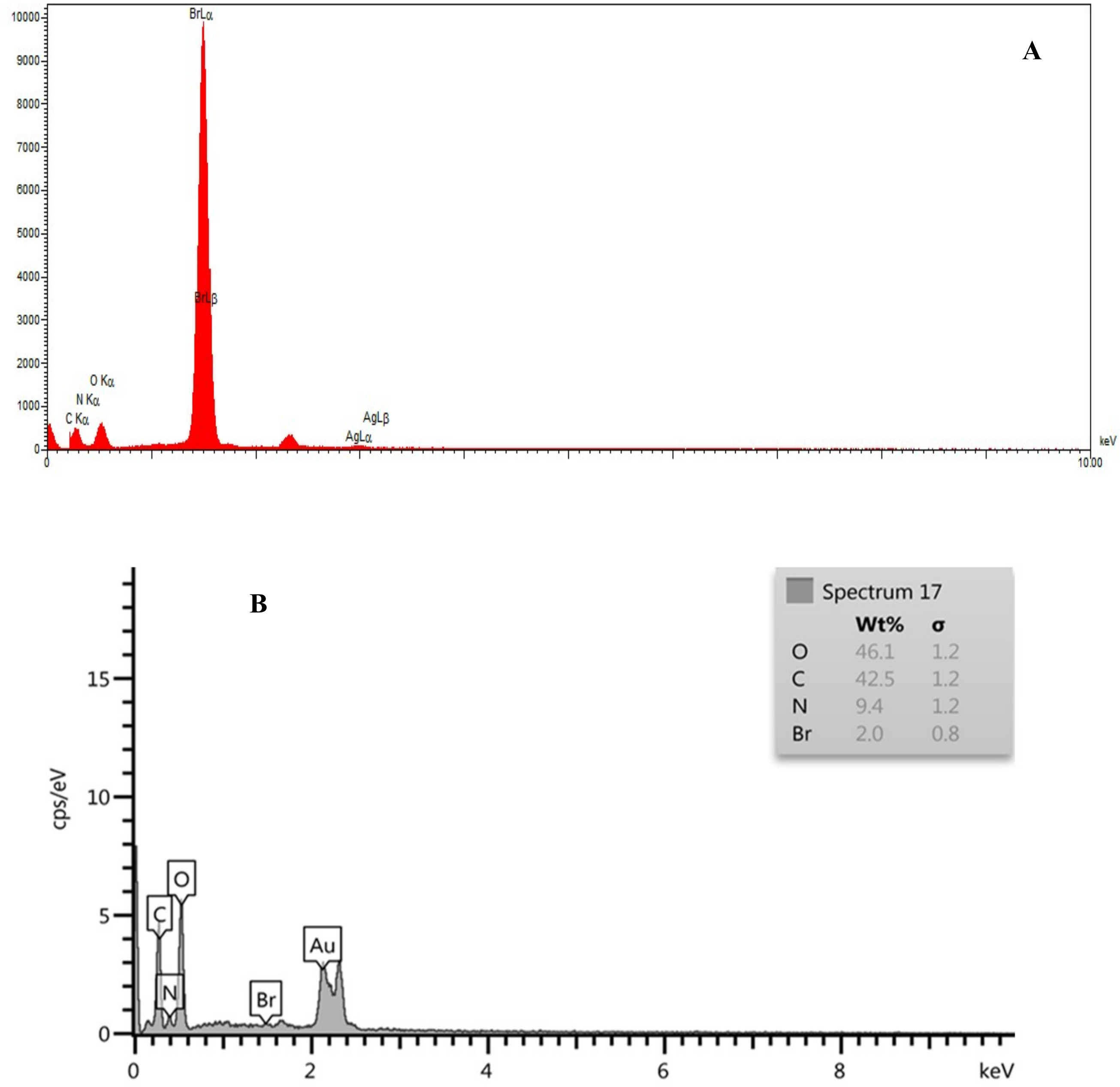

Fig. 6 EDX spectrum of neomycin nanoparticles: (A) Before CTAB removal, (B) After CTAB removal.

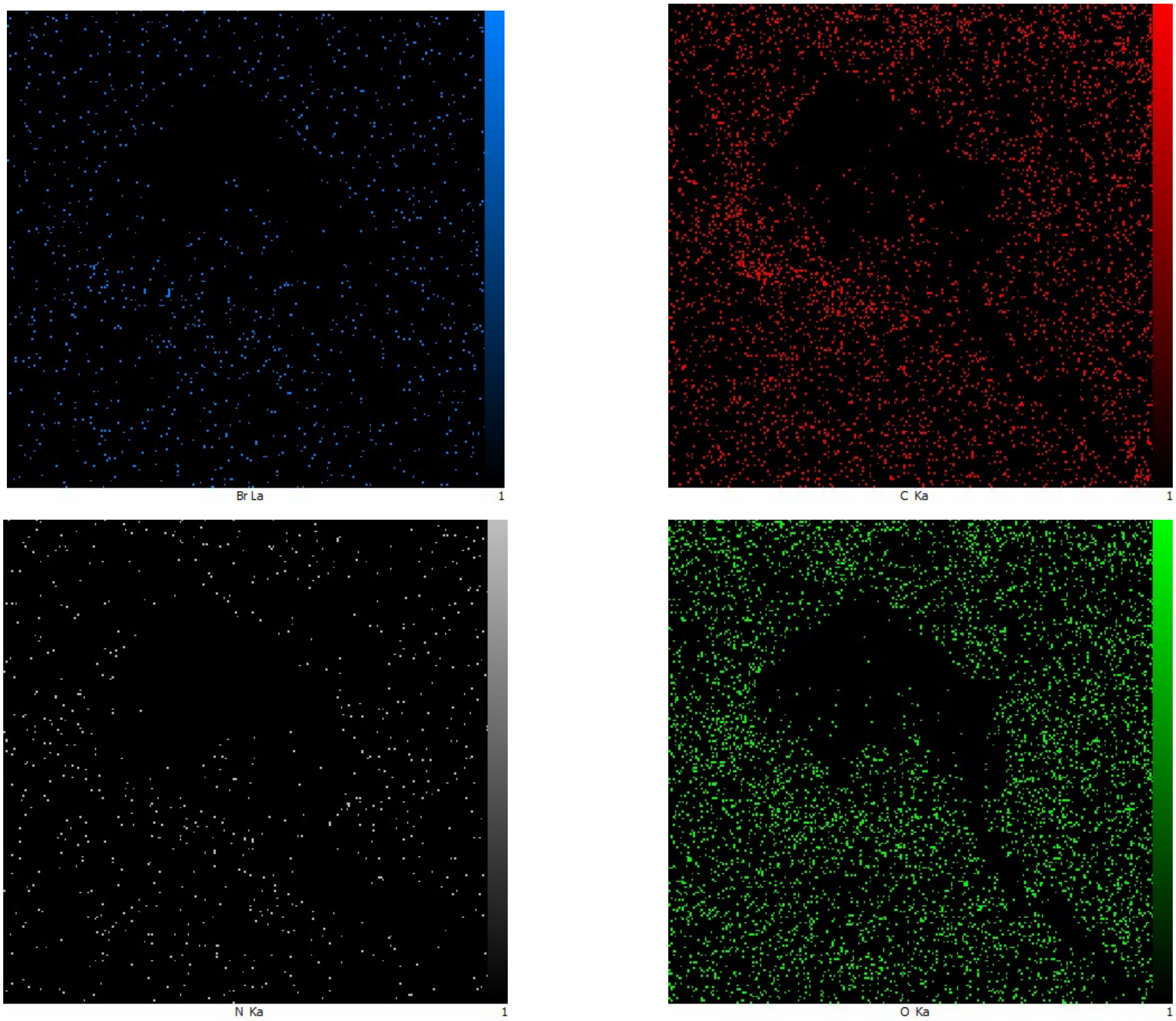
Br La
1
C Ka
1
N Ka
1
O Ka
1

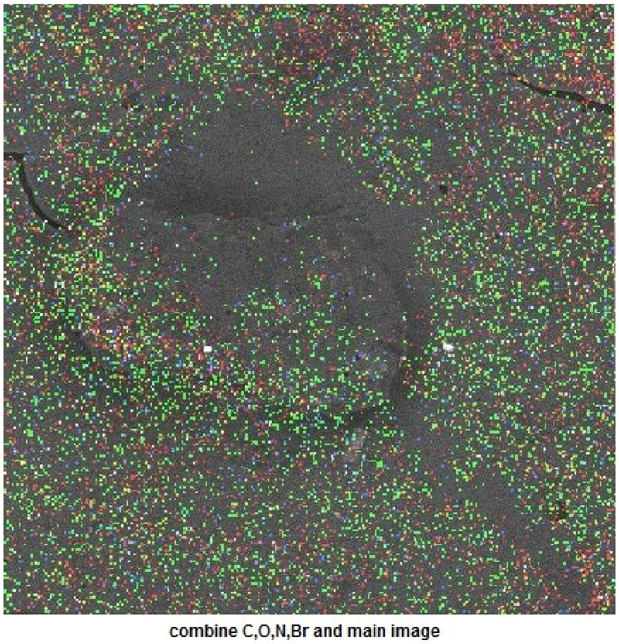

Fig. 7 Elemental mapping of synthesized neomycin.

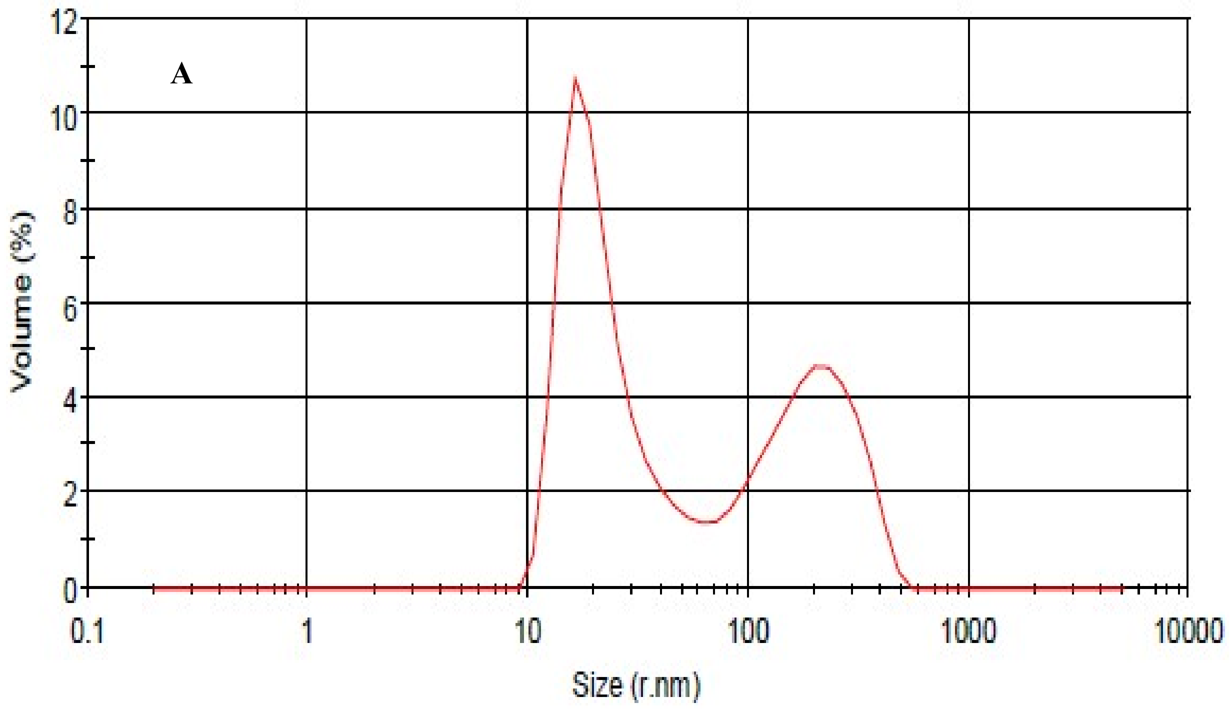

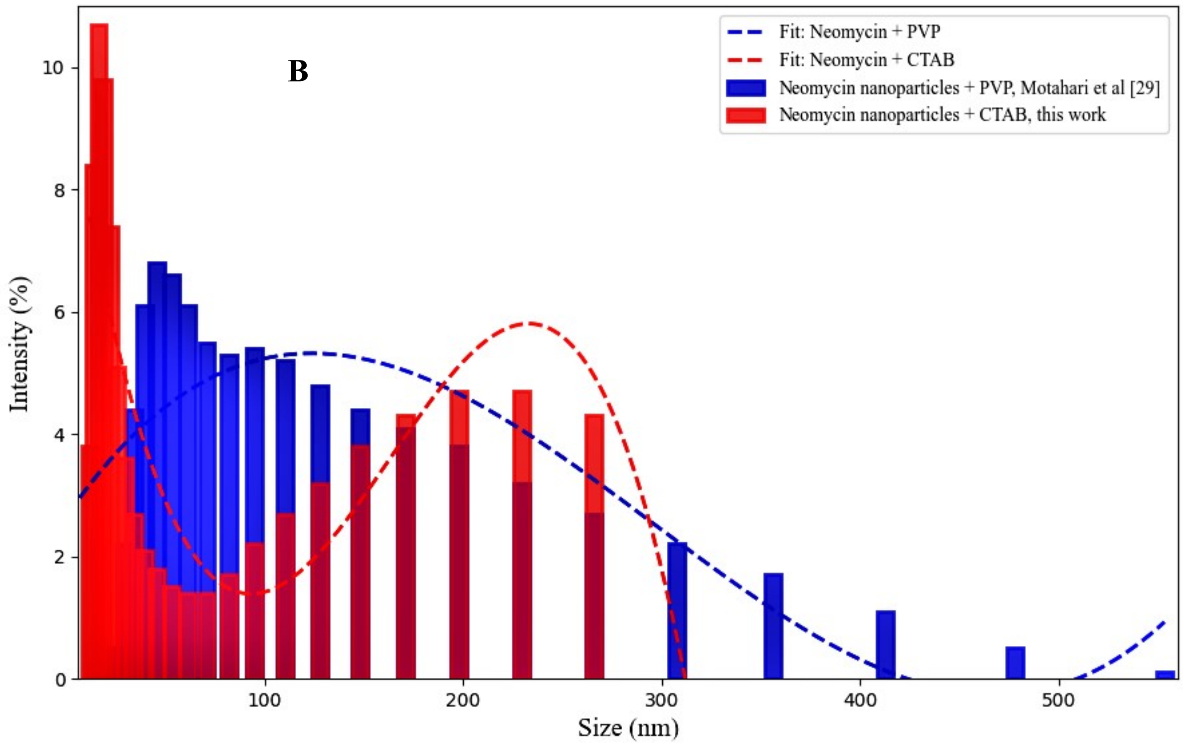

Fig.8 DLS diagram (A) and size distribution (B) for synthesized neomycin nanoparticles.

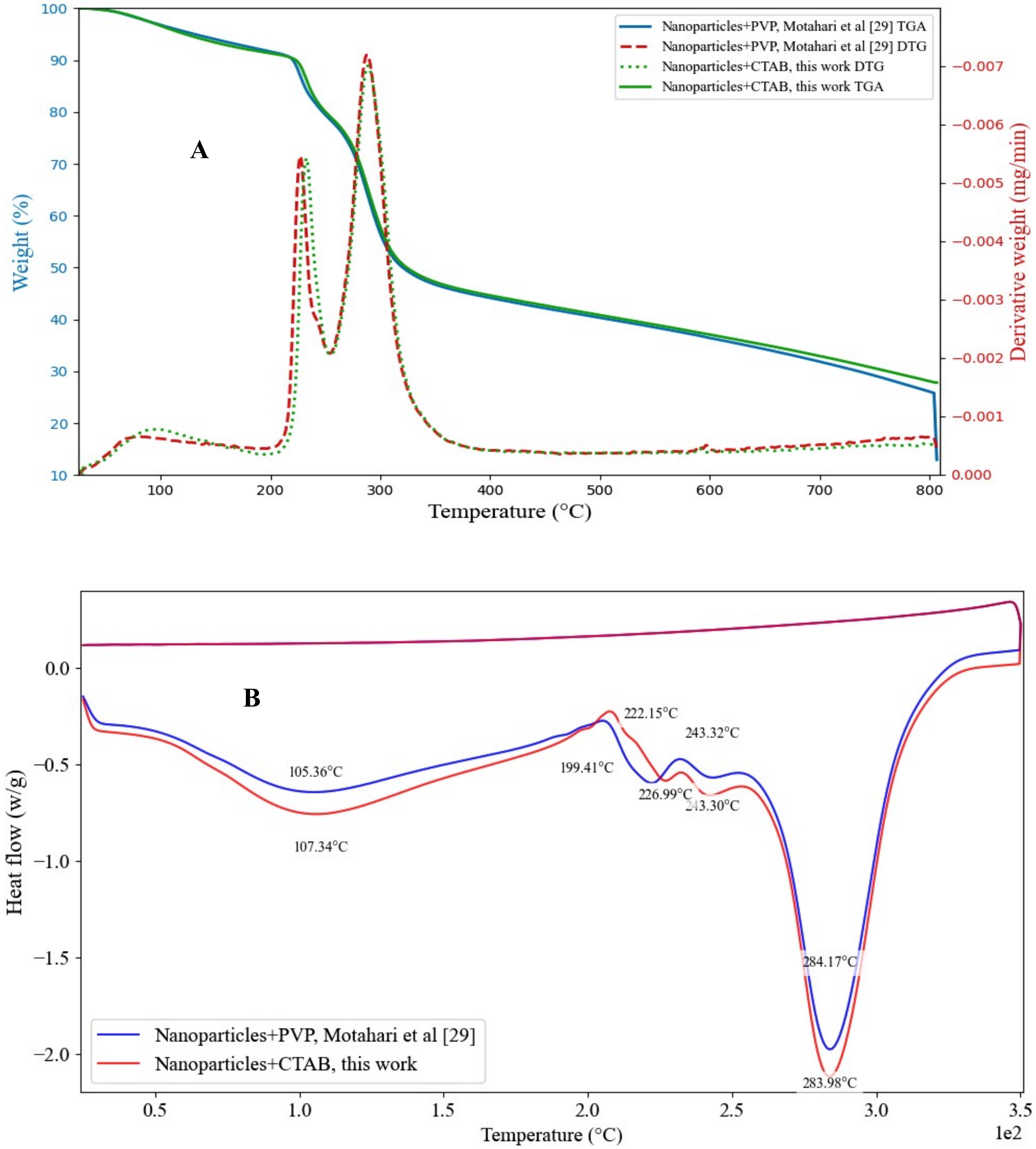

Fig.9. TGA (A), DSC (B) spectra for synthesized neomycin nanoparticles in the presence of CTAB and and comparison with existing results.

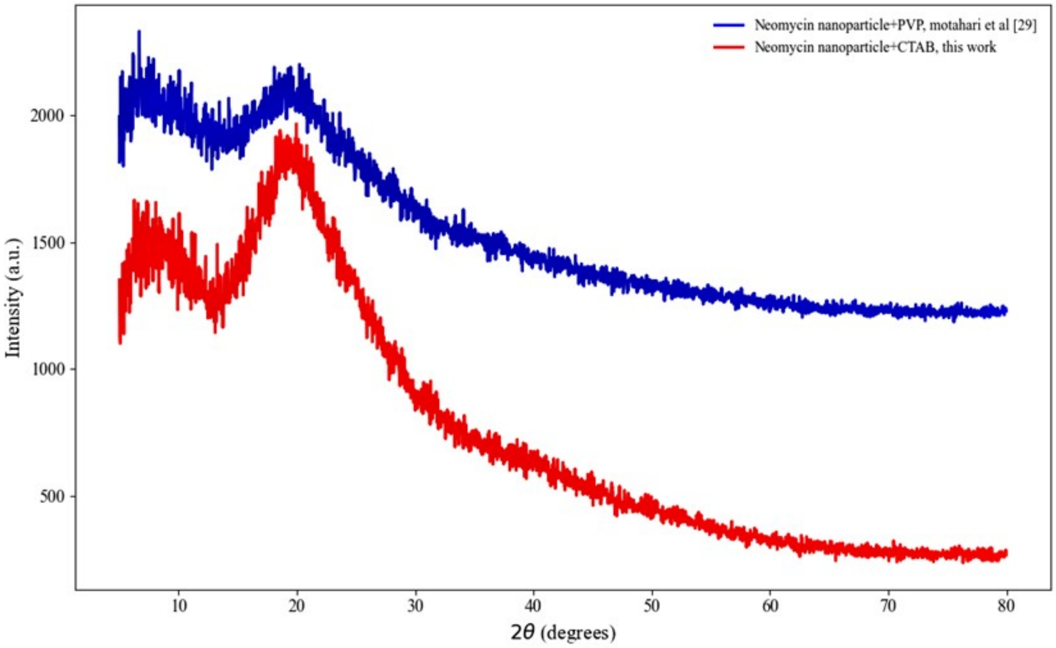

Fig. 10. XRD spectra for the synthesized neomycin nanoparticles in the presence of CTAB and comparison with existing results.

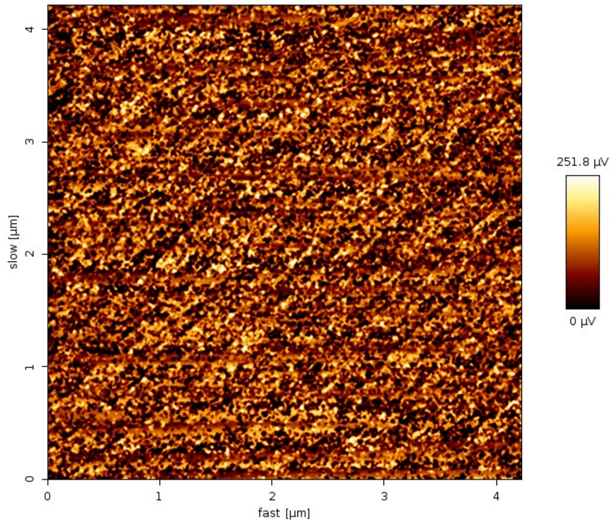
slow [µm]
fast [µm]
251.8 µV
0 µV

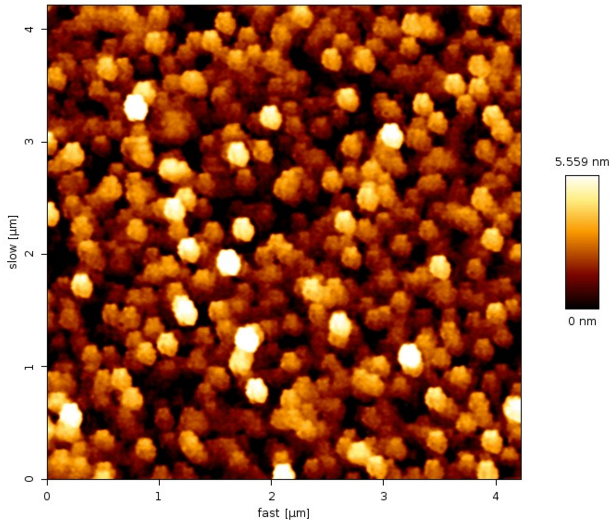
slow [µm]
fast [µm]
5.559 nm
0 nm

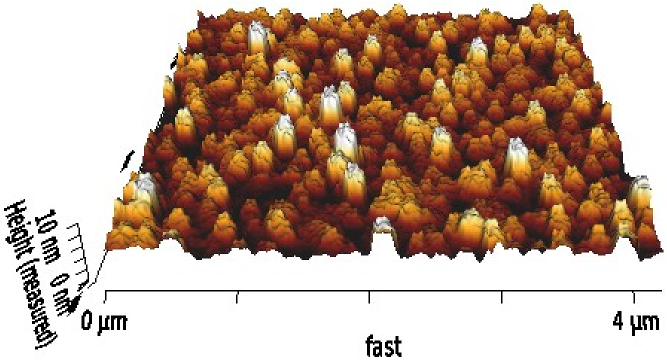
10 nm
0 nm
Height (measured)
0 µm
4 µm
fast

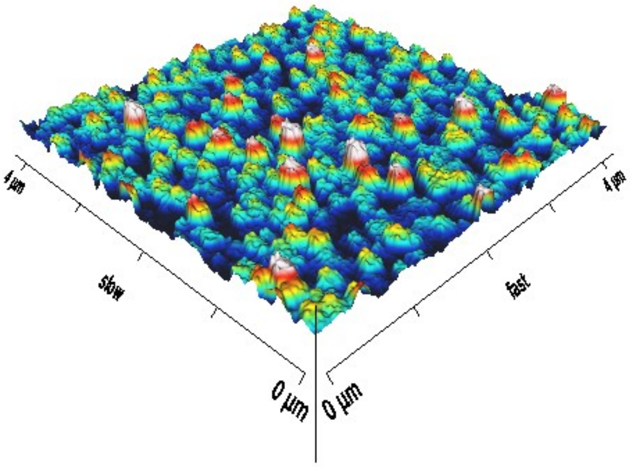
4 µm
slow
0 µm
0 µm
fast
4 µm

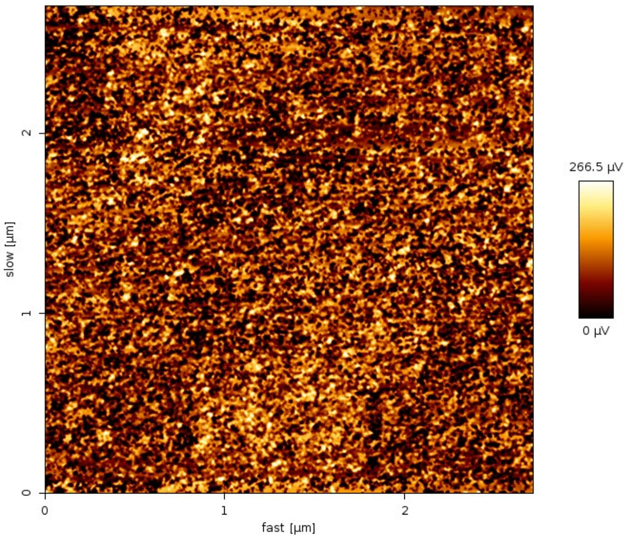
slow [µm]
fast [µm]
266.5 µV
0 µV

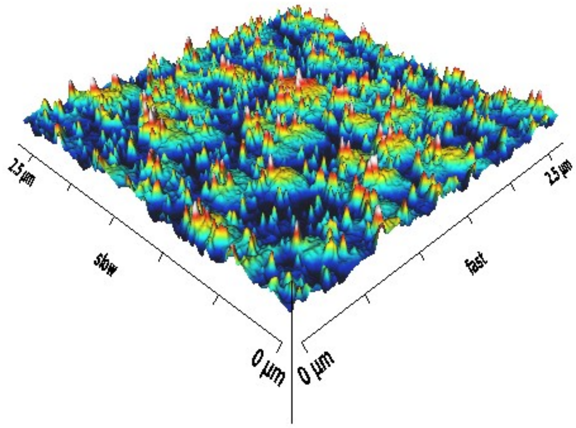
2.5 µm
slow
0 µm
0 µm
fast
2.5 µm

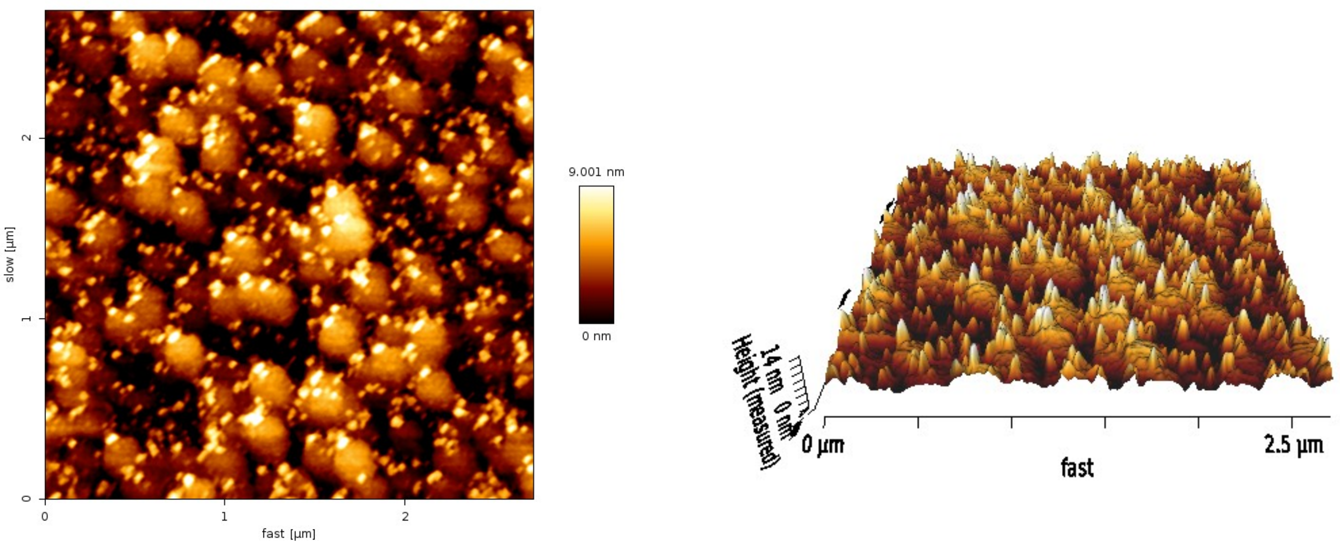

Fig. 11 AFM images of the neomycin nanoparticles.

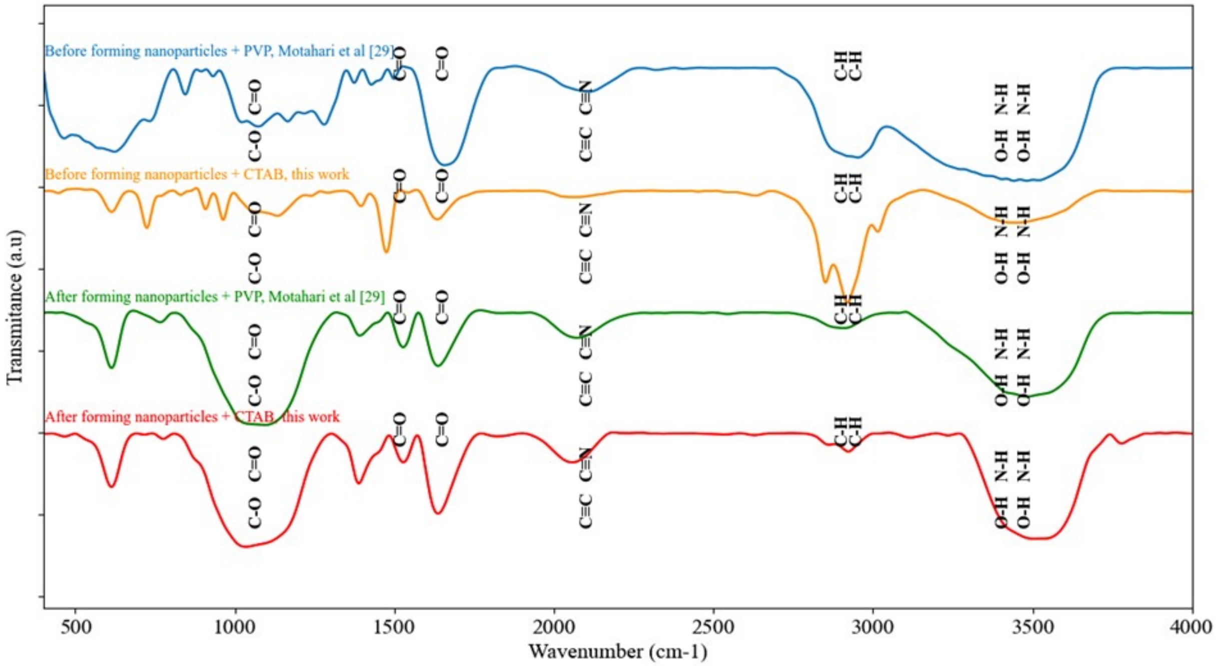

Fig.12 FT-IR spectroscopy before and after neomycin nanoparticles formation in the presence of CTAB and comparison with existing results.

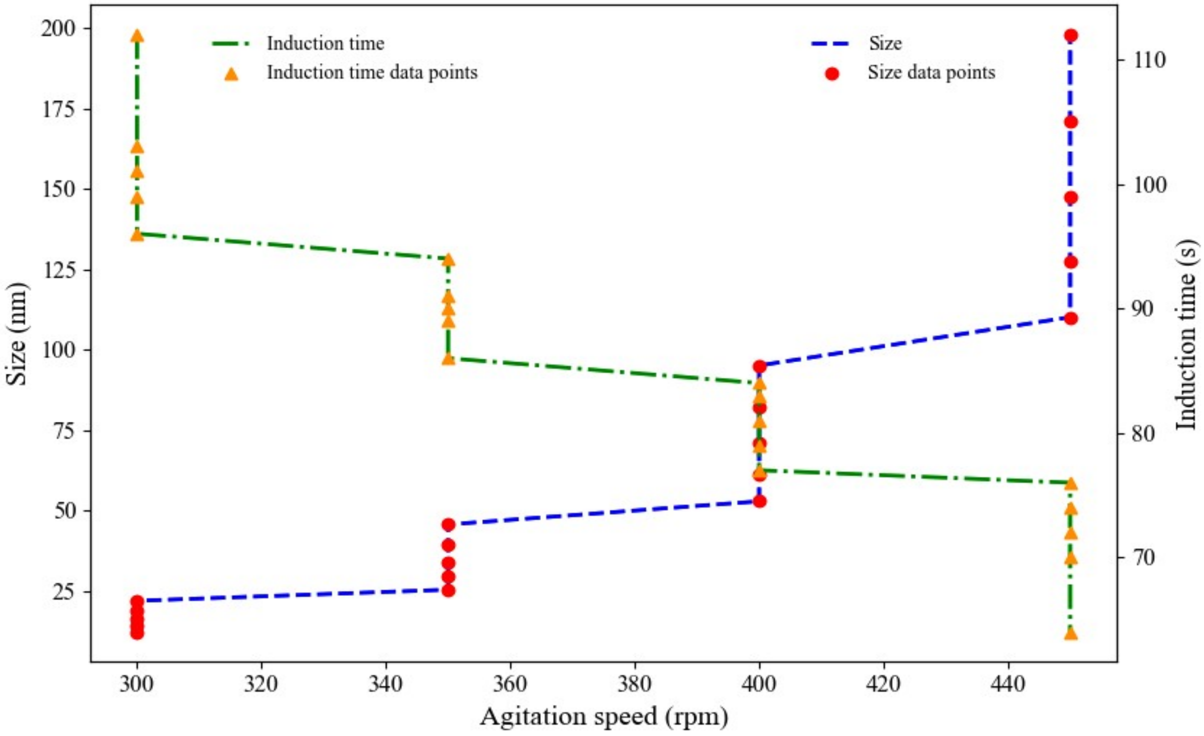

Fig. 13 Effect of agitation rate on induction time and size of neomycin nanoparticles in the presence CTAB.

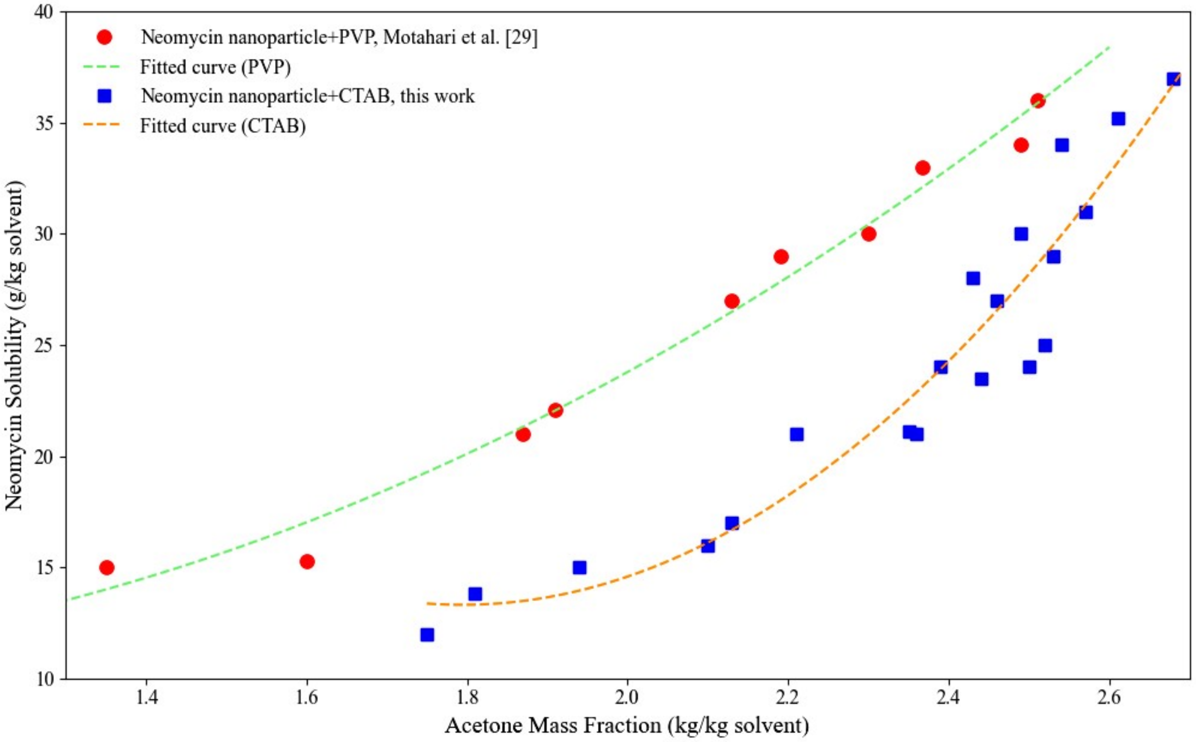

Fig.14 Neomycin solubility versus acetone mass fraction in the presence of CTAB and comparison with existing results.

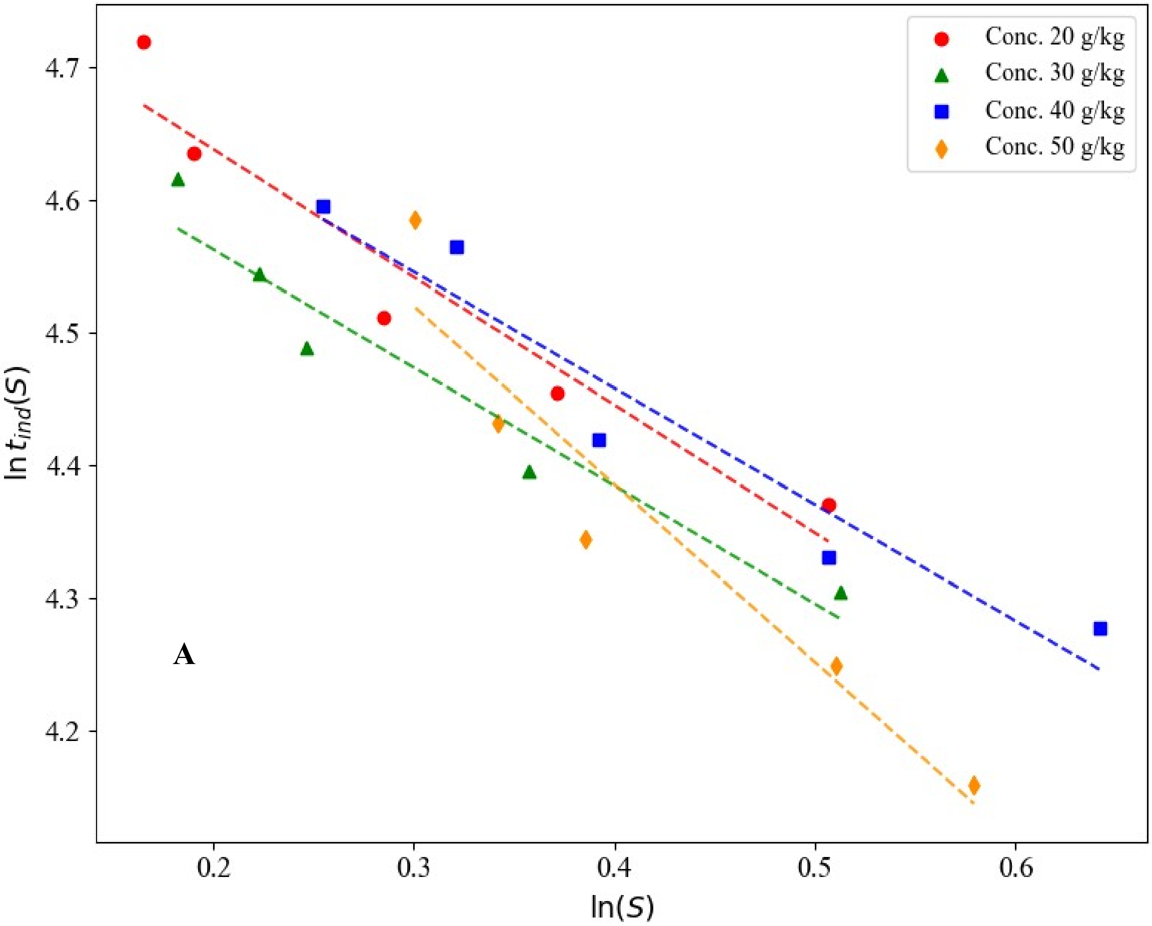

Conc. 20 g/kg
Conc. 30 g/kg
Conc. 40 g/kg
Conc. 50 g/kg
4.7
4.6
4.5
4.4
4.3
4.2
$\ln t_{ind}(S)$
A
0.2
0.3
0.4
0.5
0.6
$\ln(S)$

232

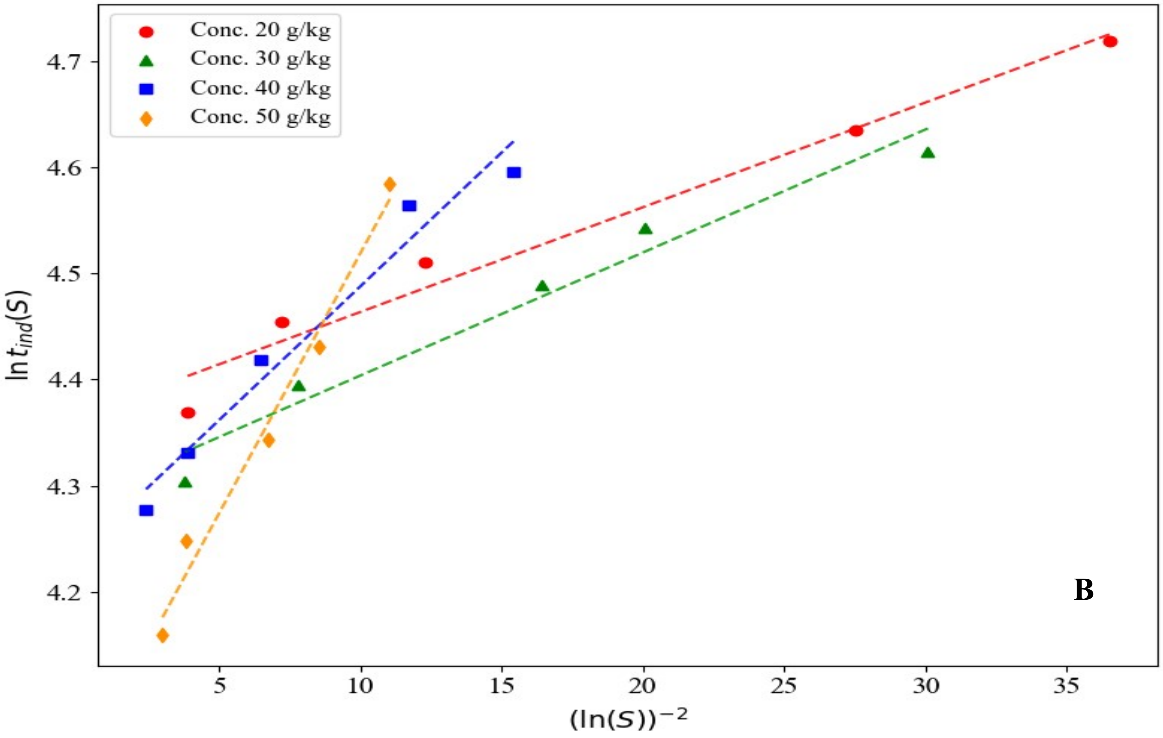

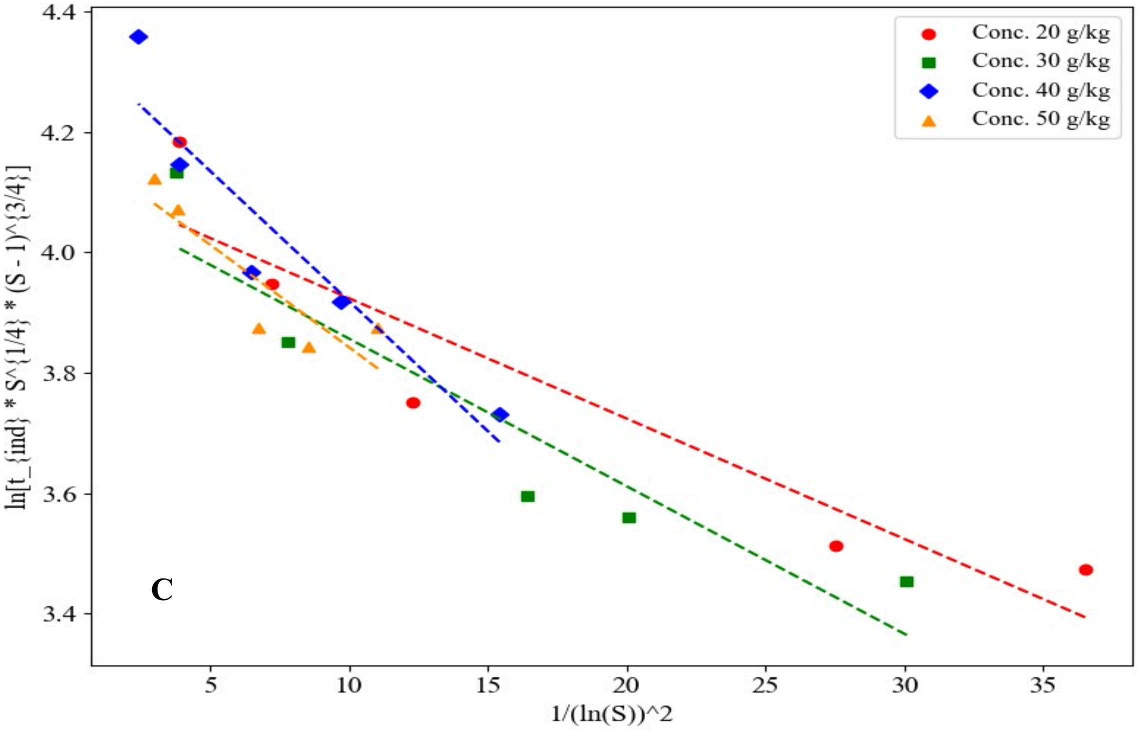

Fig.15 (A) ln tind v.s $1/(\ln S)^2$ and (B) ln tind v.s ln S and (C) ln(t_ind.S^(1/4)(S-1)^(3/4)) versus $1/(\ln(S))^2$ at temperatures of 25 °C and the initial concentration of 0.6 g/kg of CTAB.

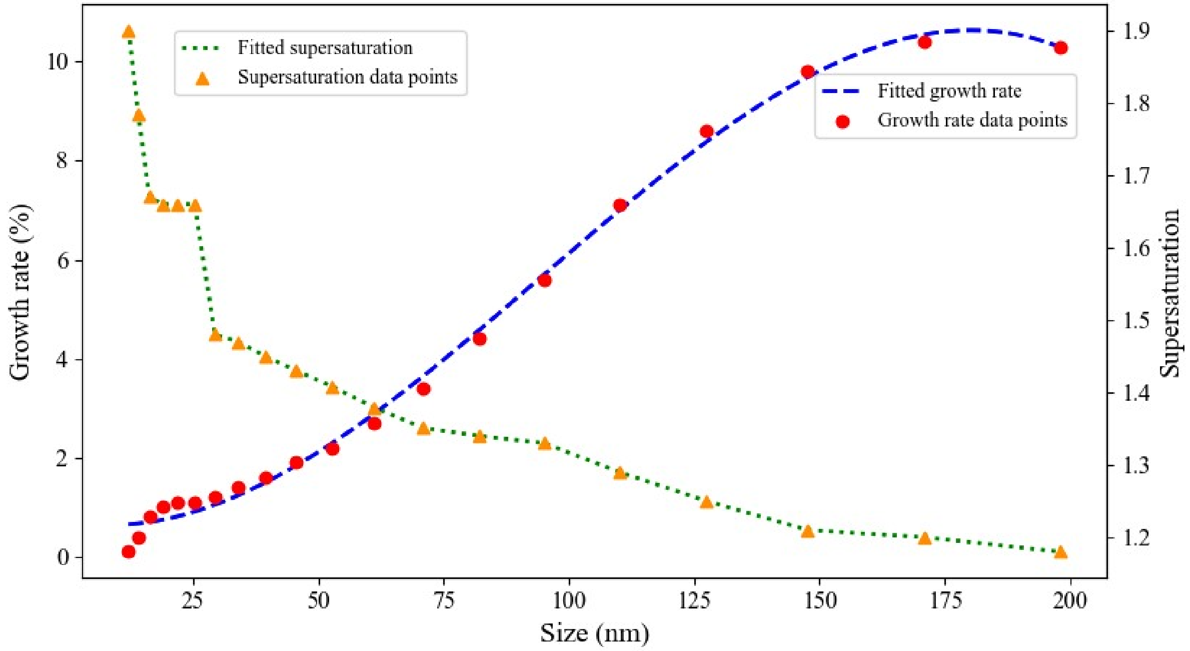

Fig. 16. Direct effect of supersaturation on the growth rate and size of neomycin nanoparticles in the presence of CTAB.

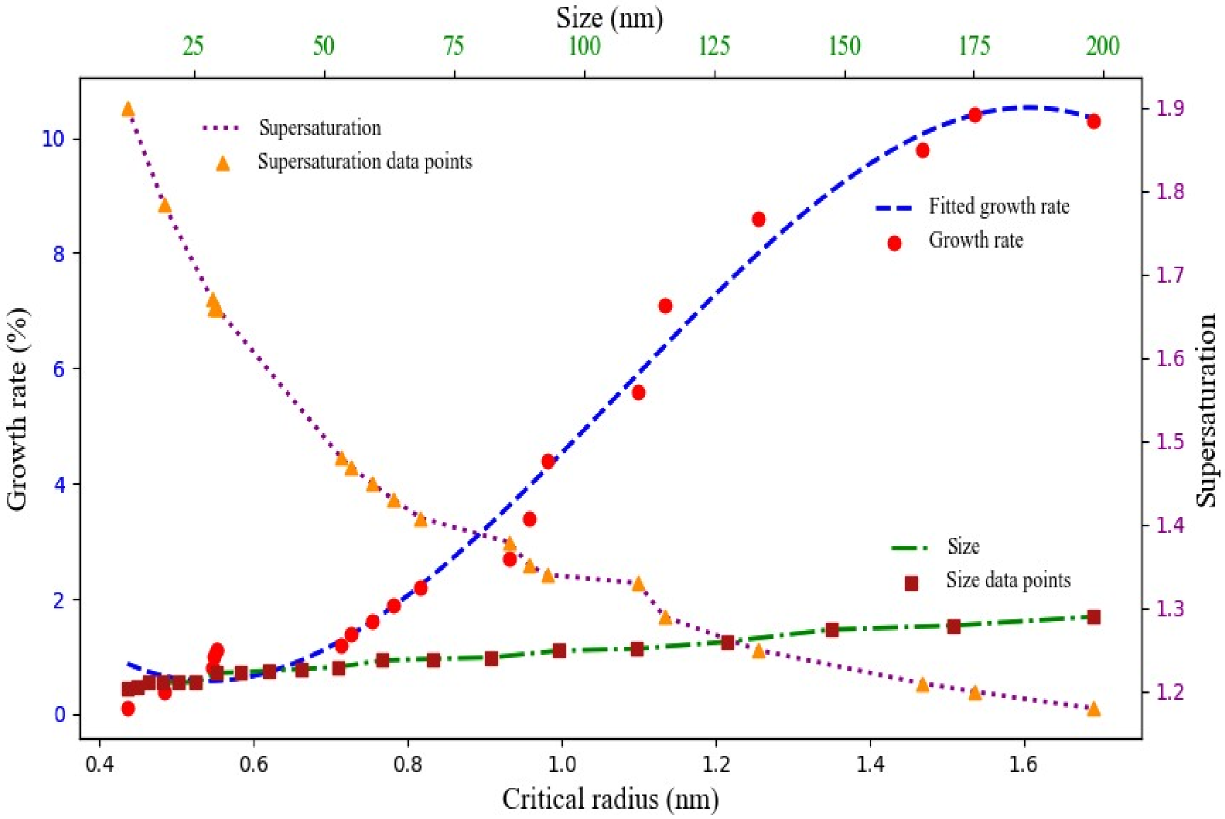

Fig. 17. Direct relationship between critical radius and supersaturation and its effect on the growth rate and size of neomycin nanoparticles in the presence of CTAB.

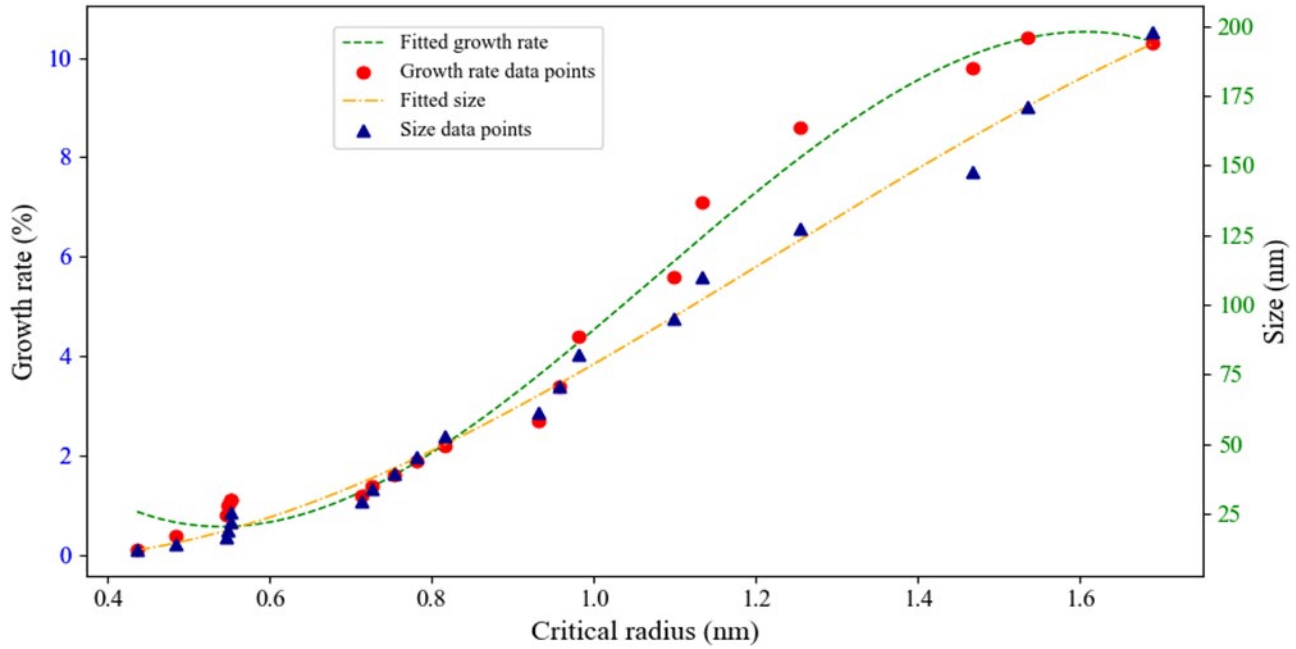

Fig.18 Effect of critical radius on the growth rate and final size of neomycin nanoparticles in the presence of CTAB stabilizer.

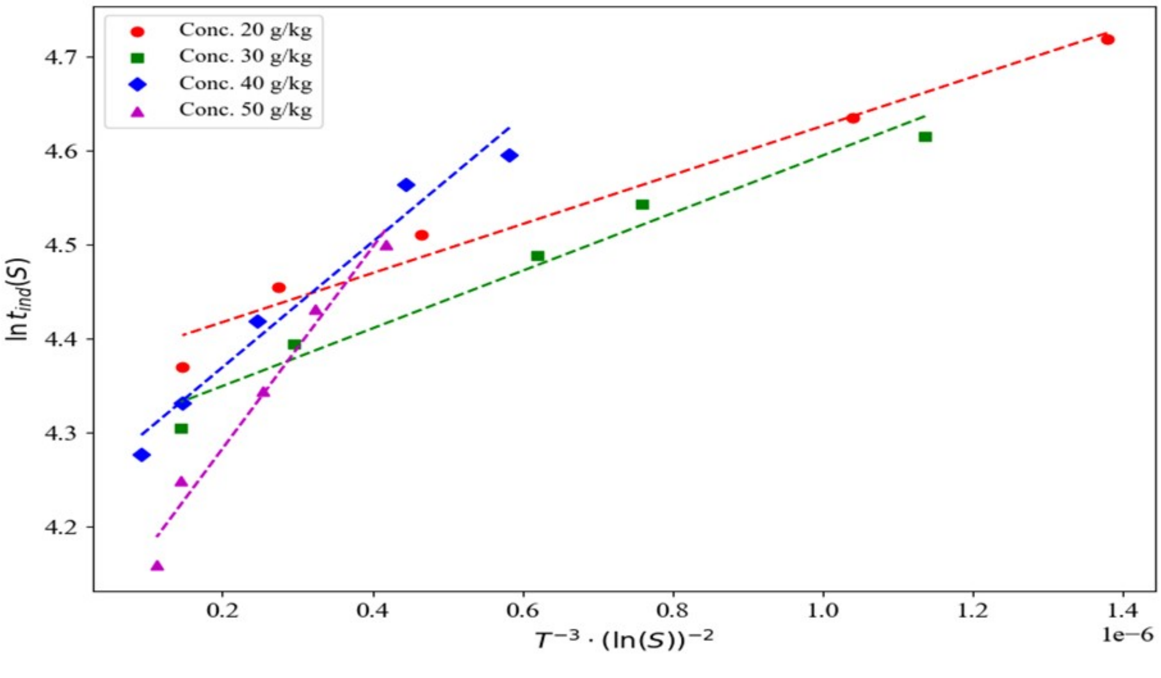

Fig.19. ln tind v.s 1/(T3 (ln S)2) for CTAB solution concentrations of 0.6 g/kg at 25 °C.

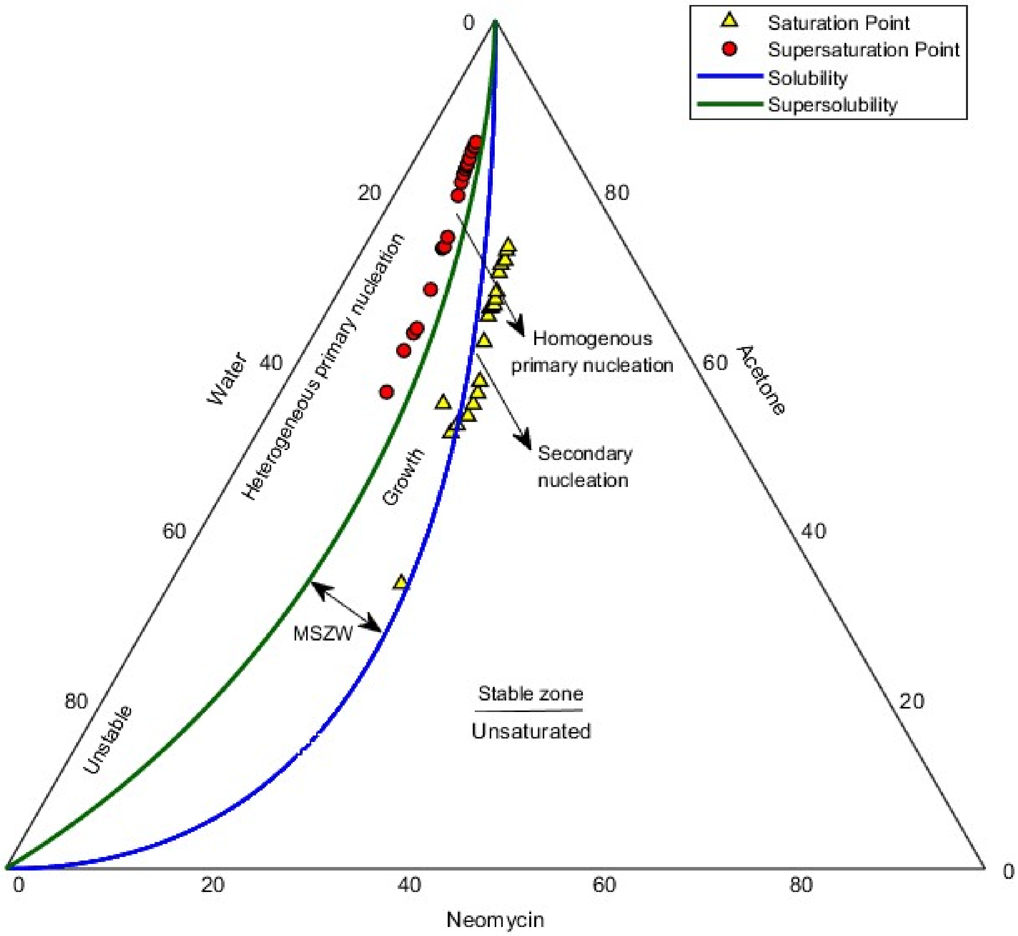

Fig.20. MSZW of neomycin as a function of weight percentage in the neomycin/water/acetone system in the presence of CTAB solution with concentrations of 0.6 g/kg.

4. Tables of manuscript

Table 1. Comparison between the induction time measurement using two methods of visual and in situ turbidimeter.

Supersaturation	Number of repeats	Induction time (s) (Visual method)	Standard deviation	Induction time (s) (Turbidimeter method)	Standard deviation
1.18	8	129	10	112	10
1.2	8	117	9	103	9
1.21	8	123	11	101	10
1.25	8	121	10	99	9
1.29	8	115	9	96	8
1.33	8	109	8	94	8
1.34	8	114	9	91	8
1.351	8	101	8	90	7
1.379	8	110	9	89	7
1.408	8	103	10	86	8
1.43	8	102	9	84	7
1.45	8	109	8	83	6
1.47	8	97	8	81	7
1.48	8	93	9	79	7
1.66	8	100	10	77	7
1.663	8	95	8	76	6
1.664	8	99	8	74	6
1.67	8	85	8	73	5
1.785	8	88	7	70	6
1.9	8	79	7	64	5

Table 2. Solubility, induction time, critical radius and supersaturation concentration at the nucleation point of neomycin sulfate in the presence of CTAB solution.

Agitation rate (rpm)	Neomycin initial concentration (g/kg)	Solubility (g/kg)	Supersaturation (S)	Induction time (s)	Critical radius (nm)
300	20	17	1.18	112	1.689
	20	16	1.21	103	1.467
	20	15	1.33	91	0.981
	20	13.8	1.45	86	0.753
	20	12	1.66	79	0.552
350	30	25	1.2	101	1.534
	30	24	1.25	94	1.254
	30	23.5	1.28	89	1.133
	30	21.1	1.43	81	0.782
	30	21	1.67	74	0.545
400	40	31	1.29	99	1.099
	40	29	1.379	96	0.957
	40	27	1.48	83	0.714
	40	24	1.66	76	0.552
	40	21	1.9	72	0.436
450	50	37	1.351	90	0.932
	50	35.2	1.408	84	0.817
	50	34	1.47	77	0.726
	50	30	1.666	70	0.548
	50	28	1.785	64	0.483

Table 3. Correlation coefficients of different nucleation models in the presence of 0.6 g/kg CTAB solution.

Initial neomycin concentration (g/Kg)	Line equation, R^2		
	Secondary nucleation model	Classical homogeneous nucleation model	Kashchiev heterogeneous nucleation model
20	-0.96X+4.83; 0.9297	0.01X+4.36; 0.9728	-0.04X+4.35; 0.8702
30	-0.89X+4.74; 0.9406	0.01X+4.29; 0.9647	-0.03X+4.18; 0.8662
40	-0.88-+4.81; 0.9275	0.03X+4.24; 0.9653	-0.02X+4.12; 0.8815
50	-1.34X+4.92; 0.9120	0.05X+4.03; 0.9818	-0.02X+4.10; 0.7703

Table 4. Comparison of interfacial energy for CTAB solution concentrations of 0.6 g/kg at 25 °C with existing results.

Initial neomycin concentration ($g/kg_{solvent}$)	R^2	Interfacial energy this work ($mJ.m^{-2}$)	Interfacial energy ($mJ.m^{-2}$), Motahari et al [6].
20	0.9728	1.33	7.68
30	0.9647	1.41	8.056
40	0.9653	1.9	-
50	0.9726	2.14	-

References

1. J. W. Mullin, Crystallization, 4th ed., Elsevier, 2001, https://doi.org/10.1016/B978-0-7506-4833-2.X5000-1.
2. J. Garside, Industrial crystallization from solution, Chem. Eng. Scien,1985. 40 (1). 3-26, doi.org/10.1016/0009-2509(85)85043-0.
3. J. Nývlt, The metastable zone width of ammonium aluminium sulphate and mechanisms of secondary nucleation, Collect. Czech. Chem. Commun, 1982. 47 (4). 1184-1188, doi.org/10.1135/cccc19821184.
4. D. Kashchiev A. Firoozabadi, Induction Time in Crystallization of Gas Hydrates, J. Cryst. Growth, 2003. 250 (3). 499–515, doi.org/10.1016/S0022-0248(02)02461-2.
5. A. K. Dehghan, M. Manteghian, Promoting effect of AlN foreign particles on crystallization of sodium sulfate decahydrate, J. Crys. Gro, 2022, 593 (12), 126754, DOI:10.1016/j.jcrysgro.2022.126754.
6. S. Motahari, A. Alamdari, M. R. Malayeri, Crystallization of neomycin nanoparticles in the presence of polyvinyl pyrrolidone (PVP), Nanoscale. Adv, 2025. 7(8). 2272–2289, doi: 10.1039/d4na01031k.
7. M. Torkian, M. Manteghian, M. Safari, Caffeine metastable zone width and induction time in anti-solvent crystallization, J. Cry. Growth, 2022, 594 (15). 126790, doi.org/10.1016/j.jcrysgro.2022.126790.
8. J. Han, J. Wang, Caffeine Crystallization Induction Time Measurements Using Laser Scattering Technique and Correlation to Surface Tension in Water and Ethanol, Chinese. J. Chem. Eng, 2010, 18(5). 767-769, doi.org/10.1016/S1004-9541(09)60127-0.
